# An ex vivo organ culture screening model revealed that low temperature conditions prevent side effects of anticancer drugs

**DOI:** 10.1038/s41598-022-06945-7

**Published:** 2022-02-23

**Authors:** Tian Tian, Kanako Miyazaki, Yuta Chiba, Keita Funada, Tomomi Yuta, Kanji Mizuta, Yao Fu, Jumpei Kawahara, Xue Han, Yuna Ando, Ami Funada, Aya Yamada, Tsutomu Iwamoto, Seiji Nakamura, Ichiro Takahashi, Satoshi Fukumoto, Keigo Yoshizaki

**Affiliations:** 1 Section of Orthodontics and Dentofacial Orthopedics, Division of Oral Health, Growth and Development, Kyushu University Faculty of Dental Science, Fukuoka, Japan; 2 Section of Pediatric Dentistry, Division of Oral Health, Growth and Development, Kyushu University Faculty of Dental Science, Fukuoka, Japan; 3 Department of Pediatric Dentistry, School and Hospital of Stomatology, Tongji University Shanghai Engineering Research Centre of Tooth Restoration and Regeneration, Shanghai, China; 4 Department of Medicine, Kyushu University School of Medicine, Fukuoka, Japan; 5 Section of Oral and Maxillofacial Oncology, Division of Maxillofacial Diagnostic and Surgical Sciences, Kyushu University Faculty of Dental Science, Fukuoka, Japan; 6 Division of Pediatric Dentistry, Department of Community Social Dentistry, Tohoku University Graduate School of Dentistry, Sendai, Japan; 7 Department of Pediatric Dentistry/Special Needs Dentistry, Division of Oral Health Sciences, Graduate School of Medical and Dental Sciences, Tokyo Medical and Dental University, Tokyo, Japan

**Keywords:** Experimental models of disease, Organogenesis

## Abstract

Development of chemotherapy has led to a high survival rate of cancer patients; however, the severe side effects of anticancer drugs, including organ hypoplasia, persist. To assume the side effect of anticancer drugs, we established a new ex vivo screening model and described a method for suppressing side effects. Cyclophosphamide (CPA) is a commonly used anticancer drug and causes severe side effects in developing organs with intensive proliferation, including the teeth and hair. Using the organ culture model, we found that treatment with CPA disturbed the growth of tooth germs by inducing DNA damage, apoptosis and suppressing cellular proliferation and differentiation. Furthermore, low temperature suppressed CPA-mediated inhibition of organ development. Our ex vivo and in vitro analysis revealed that low temperature impeded Rb phosphorylation and caused cell cycle arrest at the G1 phase during CPA treatment. This can prevent the CPA-mediated cell damage of DNA replication caused by the cross-linking reaction of CPA. Our findings suggest that the side effects of anticancer drugs on organ development can be avoided by maintaining the internal environment under low temperature.

## Introduction

Currently, the side effect of chemotherapy on organ development is mainly tested by intraperitoneal injection of drugs in pregnant mice^1–4^. However, in this conventional method, the assessment of the drugs’ direct effects on these organs can be difficult. An organ culture method is commonly used to examine the direct effects of drugs on organogenesis. We previously established an ex vivo organ culture system of tooth germ using cell culture inserts^5^. Our system allows us to examine the effects of drugs on organogenesis easily by directly adding compounds into the culture medium. Therefore, we hypothesized that the ex vivo organ culture method would be useful for drug screening and searched for rescue factors in various organs.

Treatment modalities of childhood cancer start with surgery and radiation therapy, followed by drug therapy using anticancer drugs. This establishment of a combination of therapeutic methods dramatically improved the cure rate of various cancers, and currently, the 5-year survival rate of childhood cancer is over 80%^6^. However, with the improved survival rate, the occurrence of side effects related to the use of anticancer drugs has become a major clinical hurdle in childhood cancer. More than two-thirds of patients who had intensive tumor treatment during their developmental period had late systemic effects, 30 years after treatment completion^7^. Clinically, leukemia, the most common childhood cancer, constitutes 30% of all pediatric cancers^8,9^. Cyclophosphamide (CPA), an alkylating agent, is often used to treat childhood leukemia. CPA shows cytotoxic effects by binding with DNA bases to form inter-strand crosslinking to prevent DNA replication^10^. Cytotoxic drugs mainly kill rapidly-dividing tumor cells; however, they also damage normal cells showing active mitosis^11^. Organs which show intensive proliferation in the formative period are easy targets for cytotoxic drugs. For this reason, teeth, salivary grands, hair, and taste buds undergo damage as side-effects of chemotherapy. Patients treated with anticancer drugs experience symptoms such as hair loss^12,13^, taste loss^14–16^, dry mouth^17^, and tooth agenesis^18,19^. Among these, tooth defects are irreparable, and this has become an important factor that reduces patients’ overall quality-of-life.

Tooth germ morphogenesis begins with the epithelium thickness at the initiation stage, and develops following the bud, cap, and bell stages^20^. During molar development, proliferative cells are present throughout the bud stage to the differentiated phases of tooth germ formation (from embryonic day (E) 13.5 to postnatal day (P) 0 in the first mouse molar) and are most abundant in the early bell stage (E16.5 in the first mouse molar). Thus, cell proliferation plays an important role in tooth morphogenesis and sizing^21,22^. These developmental stages are involved in tolerance to drugs^3^. The average onset age of childhood leukemia is 2–5 years, with significant side effects in teeth development, especially in patients aged < 3 years. In human tooth development, calcification of most permanent teeth begins within the first year of birth, whereas the onset of calcification of premolars and second molars begins at a slightly later time, in the second or third year of birth. Therefore, in childhood cancer survivors, hypoplasia of the teeth is most common in those teeth. In addition, early developing teeth have more root than crown hypoplasia, compared to late developing teeth. These phenomena are consistent with the process, timing, and sequence of tooth development^23^.

New comprehensive methods such as molecular targeted therapy and immunotherapy have emerged in the field of cancer treatment. However, chemotherapy is still the required and even the first choice treatment for most patients. Therefore, exploring how to avoid the side effects of chemotherapy is crucial. Cryotherapy, which induces vasoconstriction due to low temperatures, is used to reduce and prevent the side effects of chemotherapy drugs. Oral cryotherapy has been shown to reduce the incidence of chemotherapy-induced mucositis by approximately 40%^4,24^. In addition, nail and peripheral nerve damage was relieved to some extent by limb cryotherapy^25^. Previous studies have shown that oral cooling cryotherapy, using ice chips, is an effective treatment for the prevention of 5-fluorouracil-induced stomatitis^26^. The blood vessels in the oral mucosa were constricted by cooling, which reduces local blood flow and thus protects the mucosa from the side effects of the medication. Osaki et al. also reported that taste dysfunction in mice caused by docetaxel administration was reduced by the subsequent drinking of ice cold water^27^. Although these approaches seem to be an effective way to suppress the side effects of chemotherapy, there are currently several limitations regarding the examination of anticancer drug effects on organ development. Furthermore, the molecular mechanisms of cryotherapy remain unknown.

In this study, we established an ex vivo ectodermal organ culture method to examine the inhibition of organogenesis by anticancer agents. We screened for side effects of anticancer drugs and examined their mechanisms using this model. Furthermore, we constructed a low-temperature system that can potentially prevent drug side effects.

## Results

### CPA interrupts tooth germ formation during the morphogenesis stage

**Figure 1:**
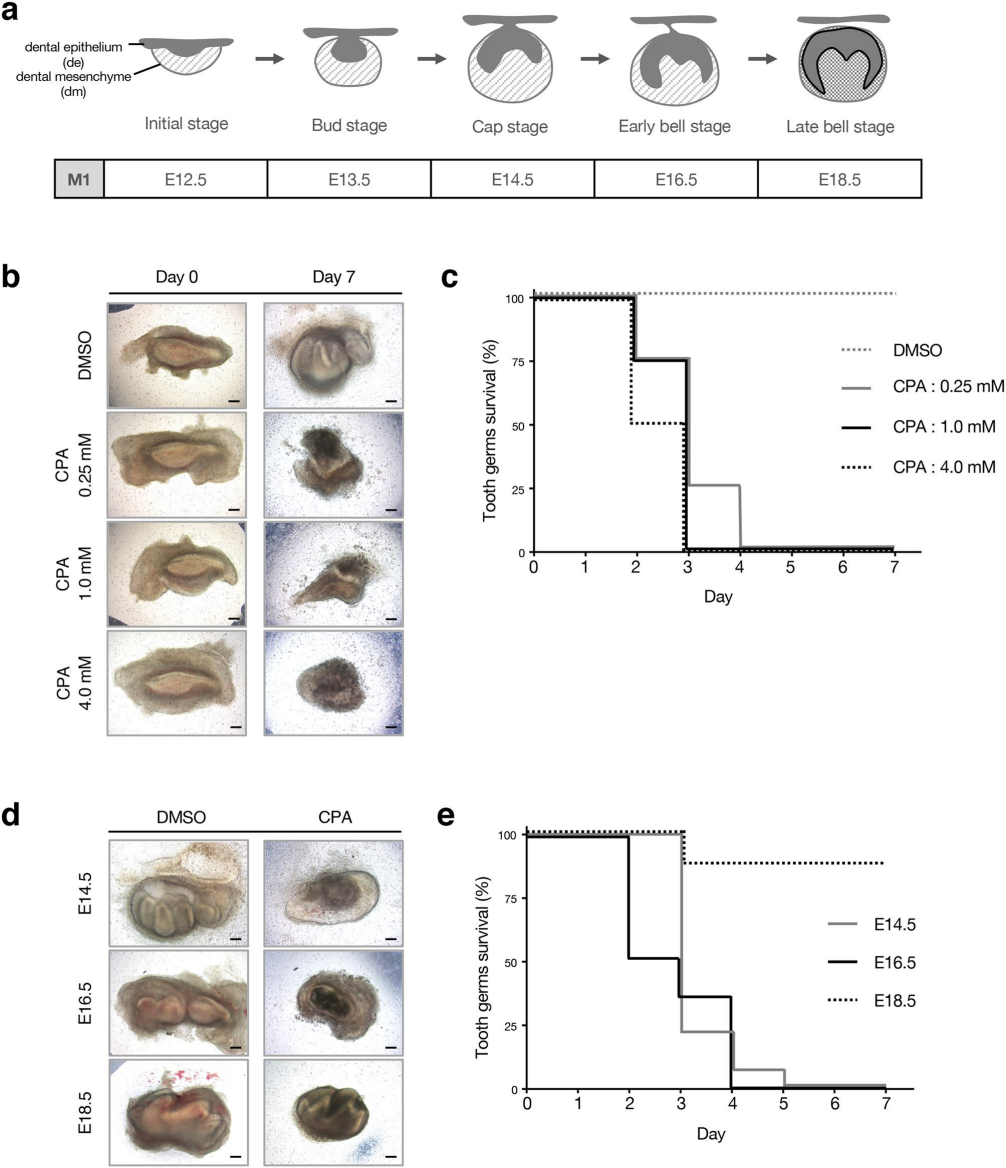
CPA interrupts the growth of tooth germ. (**a**) Schematic diagram of a mouse mandibular first molar at various stages of development. (**b**) Morphology of E14.5 tooth germs (left panel) and tooth germs cultured for seven days (right panel) in an organ culture system, treated with DMSO or CPA (0.25 mM, 1.0 mM, 4.0 mM) (n = 10). (**c**) Survival rate at various concentrations of CPA ranging between 0 and 4.0 mM. E14.5 tooth germs were dissected and different concentrations of CPA or DMSO were added in the culturing medium. (**d**) Morphology of tooth germs at different developmental stages cultured for seven days in an organ culture system, treated with DMSO or 0.25 mM of CPA (n = 8). (**e**) Survival rate of tooth germs at different developmental stages (E14.5, E16.5, E18.5), treated with 0.25 mM of CPA. Scale bars 200 µm.

Tooth development processes were divided into five stages (initiation, bud, cap, early bell, and late bell), based on the shape of the developing tooth germ (Fig. 1a). We first examined whether treatment with CPA has effects on the growth of tooth germ in an organ culture system. E14.5 mandibular tooth germs were dissected and then treated with DMSO or CPA at 0.25 mM, 1.0 mM, or 4.0 mM (Fig. 1b). After 2 days of CPA treatment, the morphological difference was observed between control and CPA-treated experimental groups. After 7 days, the growth of all tooth germs in the CPA groups (0.25 mM, 1.0 mM, and 4.0 mM) were inhibited, and no tooth structure was formed (survived tooth; n = 0/10, per group); however, it formed in the control group (survived tooth; n = 10/10) (Fig. 1b,c, and Supplementary Fig. S1a). To verify CPA effects on tooth germs at different developmental stages, E14.5, E16.5, and E18.5 mandibular tooth germs were dissected and then treated with DMSO or CPA (0.25 mM). After 7-days of CPA treatment, the growth of E14.5 and E16.5 tooth germs were completely inhibited (survived tooth; n = 0/8, per group) (Fig. 1d,e, Supplementary Fig. S1b). However, the E18.5 CPA-treated group showed a higher survival rate (survived tooth; n = 7/8 in the E18.5 CPA group) than other CPA-treated groups and formed tooth germ structure (Fig. 1d,e, Supplementary Fig. S1b). These results suggest that CPA interrupts the development and formation of tooth germs during the period of morphogenesis.

### Treatment with CPA suppresses proliferation and induces apoptosis in tooth germ

**Figure 2:**
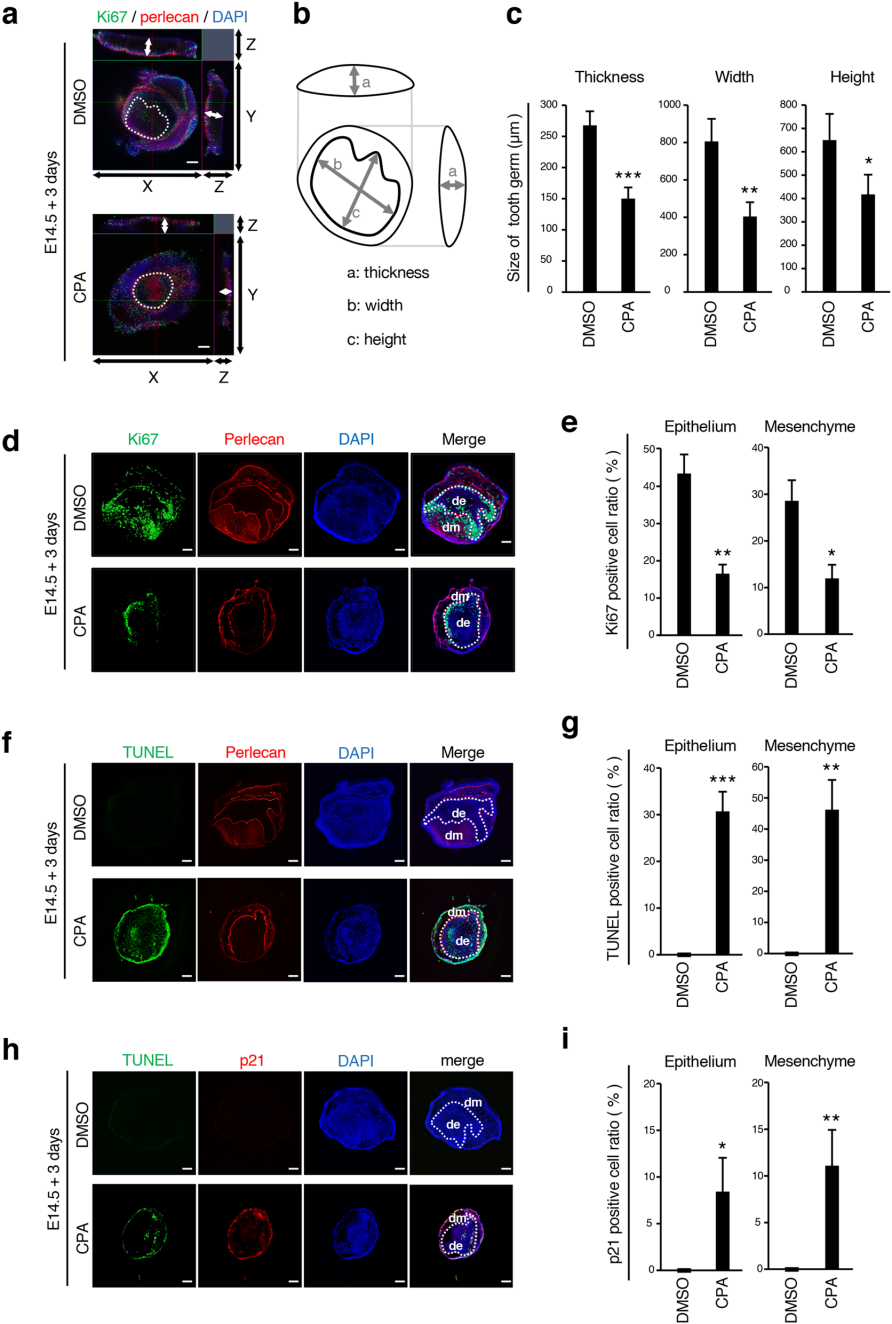
CPA inhibits cell proliferation and induces cell apoptosis in tooth germ. (**a**) Whole-mount immunohistochemistry of Ki67 (green), Perlecan (red) in E14.5 tooth germs cultured for three days, treated with DMSO or 0.25 mM CPA. Perlecan is used as a marker of basement membranes. White lines represent basement membranes of teeth. White arrows indicate thickness of tooth germ, observed from transverse and longitudinal sections. (**b**) Schematic representation of the method used for measuring the size of cultured tooth germs. (**c**) Quantified size of tooth germ E14.5 tooth germs cultured for three days, treated with DMSO or 0.25 mM CPA. Thicknesses, widths and heights of tooth germs were measured at the thickest part of whole-mount sections by ImageJ software and averaged from three different samples of each group (n = 3). (**d**) Immunofluorescence of Ki67 (green), Perlecan (red) in the section of E14.5 tooth germs cultured for three-day treated with DMSO or 0.25 mM of CPA. Nuclei were stained with DAPI (blue). Perlecan is used as a marker of basement membranes. *De* dental epithelium, *dm* dental mesenchyme. (**e**) Quantification of the Ki67-positive cell ratio in the epithelium and mesenchyme, treated with DMSO or 0.25 mM CPA (n = 4). The ratio was calculated as Ki67-positive cells/DAPI-stained nuclei. (**f**) TUNEL staining (green) and immunofluorescence of Perlecan (red) in E14.5 tooth germs cultured for three-day treated with DMSO or 0.25 mM of CPA. Apoptotic cells were detected by TUNEL staining (green). (**g**) Quantification of the TUNEL-positive cell ratio in the epithelium and mesenchyme, treated with DMSO or 0.25 mM CPA (n = 4). TUNEL-positive cell ratio was calculated as TUNEL-positive cells/DAPI-stained nuclei. (**h**) TUNEL staining (green) and immunofluorescence of p21 (red) in E14.5 tooth germs cultured for three days, treated with DMSO or 0.25 mM of CPA. (**i**) Quantification of the p21-positive cell ratio in the epithelium and mesenchyme, treated with DMSO or 0.25 mM CPA (n = 4). P21-positive cell ratio was calculated as p21-positive cells/DAPI-stained nuclei. Scale bars 200 µm. *p < 0.05; **p < 0.01; ***p < 0.001. Error bars represent the mean ± SD.

We next investigated the morphological changes of tooth germs by CPA treatment. Whole-mount immunohistochemistry was performed to present three-dimensional structures (Fig. 2a). Using the three-dimensional construction of confocal microscopy images, we measured the size (thickness, width, and height) of cultured tooth germ (Fig. 2b). The thickness, width, and height of tooth germs in the CPA-treated group was substantially decreased in transverse and longitudinal sections (Fig. 2c). To analyze the mechanism of CPA-mediated inhibition of tooth germ growth, immunofluorescence staining of frozen section was performed. Immunofluorescence staining of Cytokeratin 14 (CK14) and Vimentin showed the localization of epithelial and mesenchymal cells, respectively (Supplementary Fig. S2). We then analyzed the expression of Ki67, a cell proliferation marker, after 3 days of organ culture with or without CPA (Fig. 2d). The number of Ki67-positive cells was significantly decreased in CPA-treated dental epithelium and mesenchyme compared with that in the DMSO control group (Fig. 2e). We also examined the CPA effect on cellular apoptosis using TUNEL labeling (Fig. 2f). TUNEL-positive cell ratio was drastically increased in the CPA-treated group, both in dental epithelium and mesenchyme (Fig. 2g). Moreover, we analyzed the expression of *p21*, a cell cycle inhibition factor, and the expression was significantly increased in the CPA-treated group (Fig. 2h,i). These results indicate that CPA treatment suppresses proliferation and induces apoptosis in dental cells with inhibition of tooth germ growth.

### CPA inhibits proliferation of dental epithelial cells

**Figure 3:**
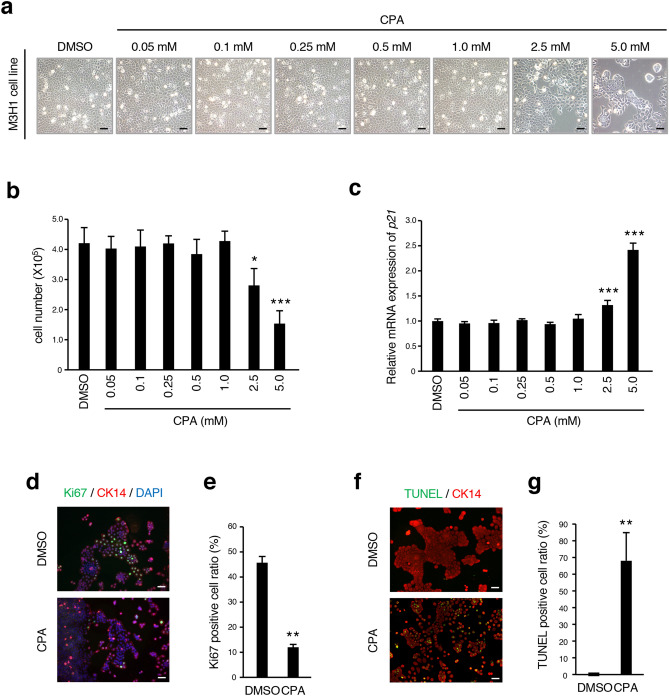
CPA suppresses proliferation and induces apoptosis of dental epithelial cells. (**a**) Phase contrast images of M3H1 cells treated with concentrations of CPA ranging between 0 and 5.0 mM. (**b**) The number of M3H1 cells treated with DMSO or CPA (concentration ranges between 0.05 and 5.0 mM) was evaluated after 72 h of culture (n = 3). (**c**) qRT-PCR analysis of the expression of p21 in M3H1 cells cultured with DMSO or CPA (between 0.05 and 5.0 mM (n = 3). (**d**) Immunocytochemistry of Ki67 (green) and CK14 (red) in M3H1 cells cultured with DMSO or 2.5 mM of CPA for 72 h on the chambers. Nuclei were stained with DAPI (blue). (**e**) The ratio of Ki67-positive cells among M3H1 cells treated with DMSO or 2.5 mM of CPA was calculated as Ki67-positive cells/DAPI-stained nuclei (n = 5). (**f**) TUNEL assay of M3H1 cells treated with 2.5 mM CPA after 72 h of culture. Apoptotic cells were detected by TUNEL staining (green). CK14-positive cells (red) were detected by immunocytochemistry and nuclei were stained with DAPI (blue). (**g**) The ratio of TUNEL-positive M3H1 cells treated with DMSO or 2.5 mM CPA was calculated as TUNEL-positive cells/DAPI-stained nuclei (n = 5). *p < 0.05; **p < 0.01; ***p < 0.001. Error bars represent the mean ± SD. Scale bars 50 µm.

We further examined CPA treatment effects on proliferation and apoptosis in dental epithelial cells using M3H1 cells. We found that the cell number in 2.5 mM or 5.0 mM CPA-treated groups was significantly reduced (Fig. 3a,b). Furthermore, the expression of *p21* was increased in 2.5 mM and 5.0 mM CPA-treated groups (Fig. 3c). We then performed immunocytochemistry staining of Ki67 and TUNEL-labeling in M3H1 cells. The number of Ki67-positive cells was reduced in CPA-treated M3H1 cells compared with the control (Fig. 3d,e). Additionally, the number of TUNEL-positive M3H1 cells was significantly increased in CPA-treated groups compared to the control (Fig. 3f,g), corresponding to ex vivo results. These findings demonstrate that CPA decreases the proliferation activity and induces cell apoptosis in dental cells.

### CPA inhibits differentiation of tooth germ and epithelial cells

**Figure 4:**
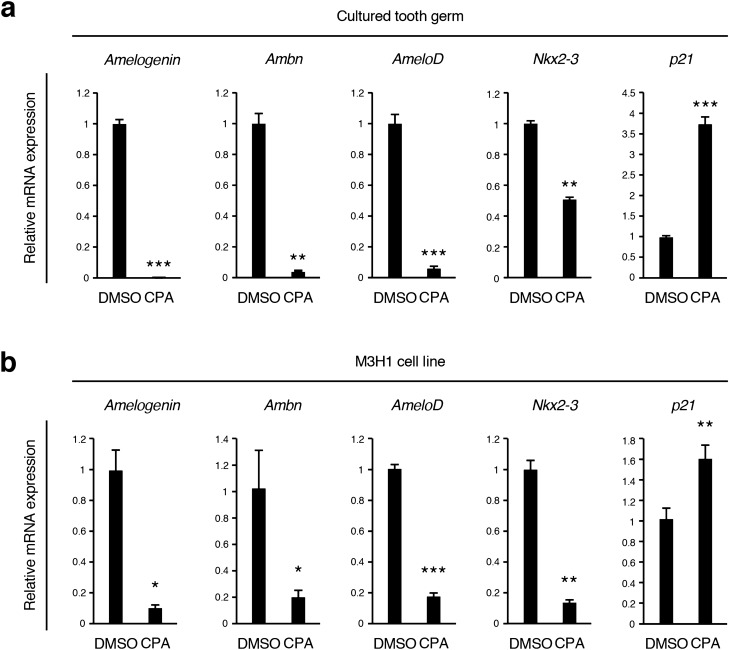
CPA inhibits differentiation of dental epithelial cells. (**a**) qRT-PCR analysis of E14.5 tooth germs treated with DMSO or 0.25 mM CPA cultured for 10 days (n = 6). Expression of *Amelogenin*, *Ambn*, *AmeloD*, *Nkx2-3*, and *p21* was normalized to *Gapdh* mRNA expression. (**b**) qRT-PCR analysis of M3H1 cells cultured with DMSO or 2.5 mM CPA (n = 4). The cells were cultured for 3 weeks, then the expressions of *Amelogenin*, *Ambn*, *AmeloD*, *Nkx2-3* and *p21* were analyzed by qRT-PCR. *p < 0.05; **p < 0.01; ***p < 0.001. Error bars represent the mean ± SD.

As CPA treatment significantly damaged tooth morphology (Fig. 1c), we tested the CPA effect on the differentiation of dental cells. We isolated total RNA from cultured tooth germ and M3H1 cells with or without CPA and performed RT-qPCR using the following marker genes: dental epithelial differentiation markers (*Amelogenin*, *Ameloblastin (Ambn)*, *AmeloD* and *Nkx2-3*)^28–30^ and *p21*. We found differentiation marker genes to be significantly downregulated in the CPA-treated group in tooth germ and M3H1 cells, while the expression of *p21* was upregulated in the CPA-treated group (Fig. 4a,b). These results suggest that treatment with CPA inhibits differentiation of tooth germ and dental epithelial cells.

### Culture at a low-temperature can prevent CPA-mediated organ damage

**Figure 5:**
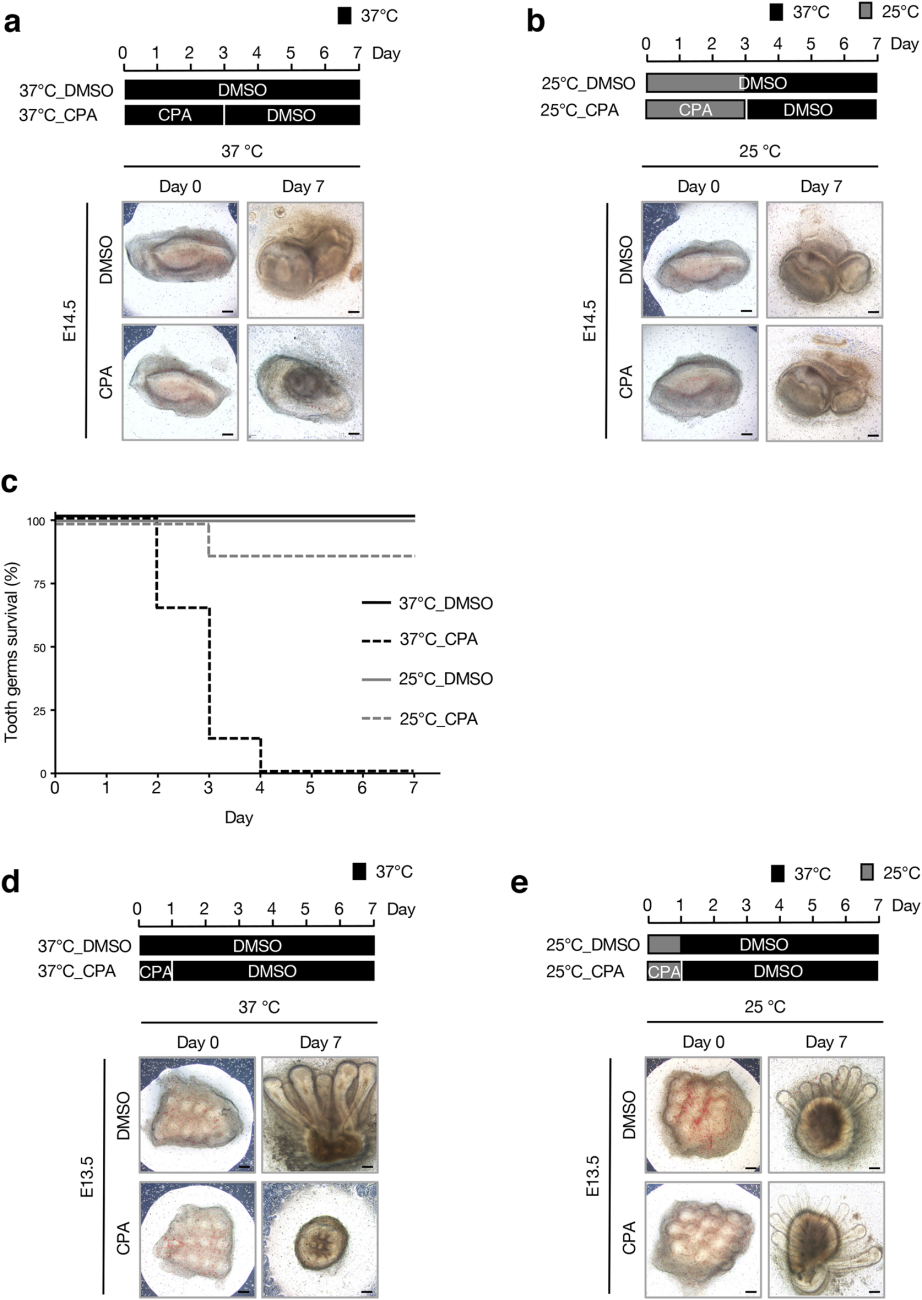
Low-temperature culture method decreases the CPA-mediated damage on the growth of cultured organs. (**a**) Morphology of tooth germs in a conventional method of organ culture. In the first three days, tooth germs were treated with DMSO or 0.25 mM of CPA. Next, culturing medium without CPA was added for four additional days. The temperature was maintained at 37 °C throughout incubation of tooth germs (n = 8). (**b**) Morphology of tooth germs at a low-temperature culture method. Tooth germs were treated with DMSO or 0.25 mM of CPA at 25 °C in the first three days. Next, culturing medium without CPA was added for four additional days at 37 °C (n = 8). (**c**) Survival rate of tooth germs under the culture conditions shown in (**a**,**b**). (**d**) Cultured E13.5 hair tissues for 7 days with the standard method (n = 8). (**e**) E13.5 hair tissues were cultured for 7 days with the low-temperature method (n = 8). Scale bars 200 µm.

To explore a preventative method of anticancer drug side effects, we attempted to rescue the tooth germs treated with CPA by adding growth factors^31,32^, which are important in tooth germ development. However, growth inhibition could not be avoided (Supplementary Fig. S3). In clinical practice, fingertip cooling is commonly used to avoid the side effects of nail peeling and skin fragility caused by chemotherapy, and has achieved certain effects^25^. Therefore, we hypothesized that CPA-mediated damage on tooth germ could be avoided by culture in low temperature. Tooth germs were cultured at different temperatures (37 °C and 25 °C, as conventional and low-temperature models, respectively). Tooth germs were treated for 3 days with or without CPA at 37 °C or 25 °C and then cultured for 4 more days without CPA at 37 °C (37°C_DMSO; 37°C_CPA; 25°C_DMSO; and 25°C_CPA, respectively) (Fig. 5a,b). Three-day treatment with CPA at 37 °C disrupted the growth of tooth germ (survived tooth; n = 0/8) (Fig. 5a). However, tooth germs cultured at 25 °C with CPA showed no evident morphological changes compared to those cultured at 25 °C without CPA (Fig. 5b). Compared to the conventional culture, tooth germs cultured at low temperature showed a higher survival rate (survived tooth; n = 7/8 in 25°C_CPA, n = 0/8 in 37°C_CPA) (Fig. 5c). We further tested this culture model using E13.5 mouse hair follicles (Fig. 5d,e). In the conventional method, growth of hair follicles was inhibited by the 1-day CPA treatment at 37 °C (Fig. 5d). While using the low-temperature culture system, growth of hair follicles was not altered by treatment with CPA (Fig. 5e). These results suggest that CPA-mediated inhibition of organ growth can be avoided at a low temperature during CPA treatment. CPA is known to be converted to 4-hydroxycyclophosphamide (4-HC) in the liver and passes through the bloodstream to act on tissues and cells^33^. To confirm whether the similar effects can be observed with active form of CPA, we conducted an experiment using 4-HC. The treatment of 4-HC inhibited the growth of tooth germs and hair follicles when cultured at 37 °C, while treatment of 4-HC at 25 °C did not disrupt development of these organs; these results are similar to that of the CPA treatment (Supplementary Fig. S4a–d).

Furthermore, we examined the effect of another alkylating agent, melphalan, on organ development using this ex vivo culture system. We found that the same effects to CPA were observed in low temperature culture method (Supplementary Fig. S5a–d). These findings indicate that the low temperature conditions can prevent not only CPA-mediated side effects but also melphalan-mediated side effects on organs.

### Low-temperature culture prevents CPA effects on proliferation, apoptosis, DNA damage, and differentiation of tooth germs

**Figure 6:**
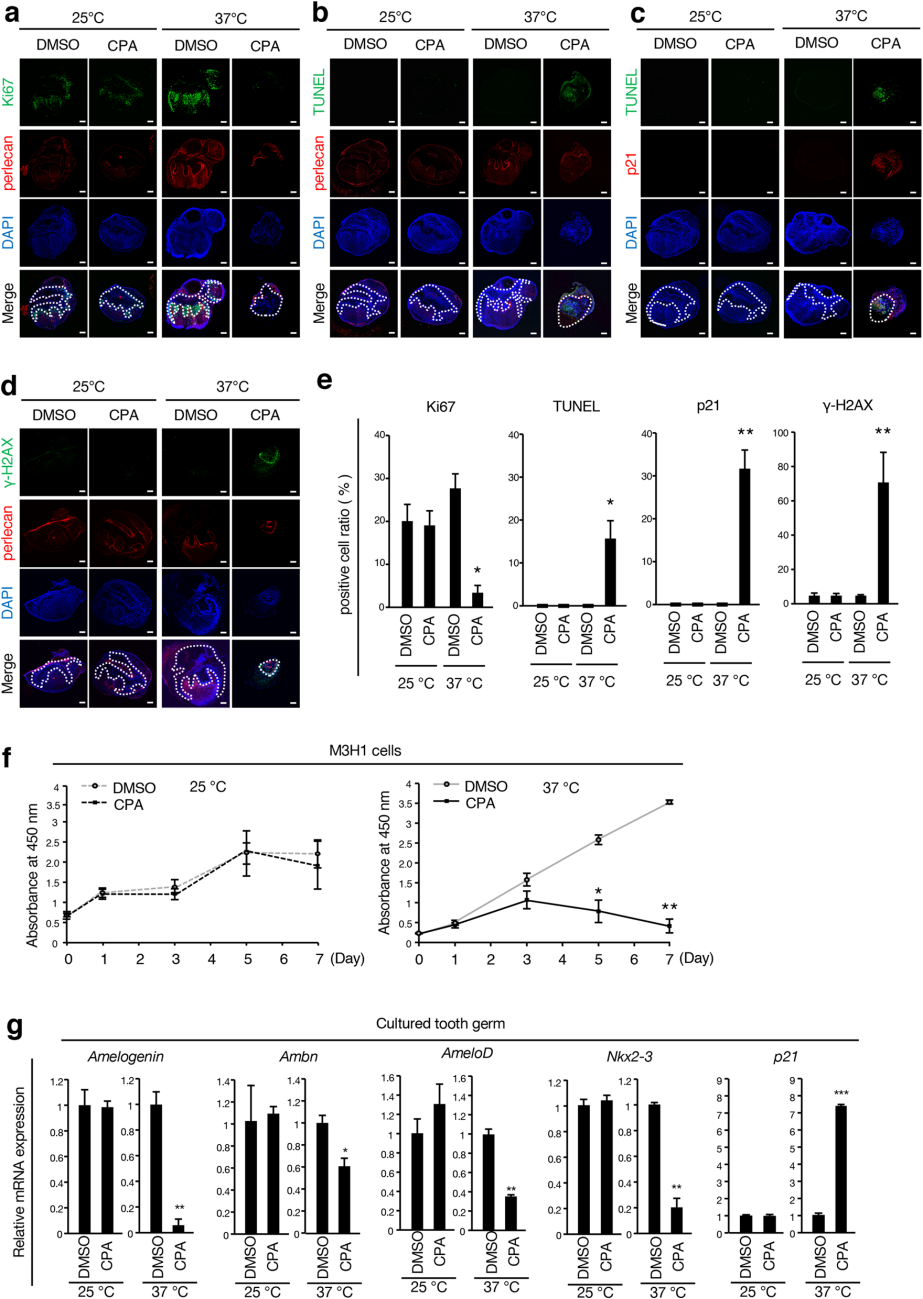
Low-temperature culture ameliorates CPA-mediated side effects on cellular proliferation, apoptosis, DNA damage, and differentiation of tooth germs. (**a**) Immunofluorescence of E14.5 tooth germs cultured for seven days in the conventional method and the low-temperature culture method with DMSO or 0.25 mM of CPA (n = 4). Ki67 (green), Perlecan (red) and nuclei were stained with DAPI (blue). Perlecan is used as a marker of basement membranes. (**b**) Apoptotic cells were detected by TUNEL (green), Perlecan (red) and DAPI (blue). (**c**) TUNEL (green), p21(red) and DAPI (blue). (**d**) DNA damage was detected by γ-H2AX(green), Perlecan (red) and DAPI (blue). (**e**) Quantified ratio of Ki67, TUNEL, p21 and γ-H2AX-positive cells in (**a**–**d**). The ratios were calculated as Ki67-positive cells (left panel), TUNEL-positive cells (middle-left panel), p21-positive cells (middle-right panel) and γ-H2AX-positive cells (right panel) /DAPI-stained nuclei. (**f**) Cell proliferation was determined by using the MTT assay. M3H1 cells treated with DMSO or 2.5 mM CPA at 25 °C or 37 °C for 7 days. (**g**) Seven-day organ culture of E14.5 tooth germs by the standard or low-temperature system (n = 6). qRT-PCR analysis of *Amelogenin*, *AmeloD*, *Nkx2-3,* and *p21* expression in cultured tooth germs after normalization to *Gapdh* mRNA expression. *p < 0.05; **p < 0.01; ***p < 0.001. Error bars represent the mean ± SD. Scale bars 200 µm.

We analyzed the effect of the low-temperature culture system on cellular proliferation, apoptosis and DNA damage by Ki67, TUNEL, *p21* and γ-H2AX immunofluorescence staining (Fig. 6a–d). After 7-day culturing of tooth germs at different temperatures with or without CPA, frozen sections of tooth germ were prepared for analysis. The ratio of Ki67-positive cells in the 25°C_CPA group was similar to that of the 25°C_DMSO or 37°C_DMSO groups (Fig. 6a,e). TUNEL and *p21*, and γ-H2AX-positive cells were observed in the 37°C_CPA group; however, they were barely detected in the 25°C_CPA group (Fig. 6b–e). Furthermore, we assessed cell viability by MTT assay in M3H1 cells (Fig. 6f). We found that the viability of cells in the CPA-treated group was significantly lower than that of the control group after 3-day culture at 37 °C (Fig. 6f, right panel); however, there was no notable difference between the two groups at 25 °C (Fig. 6f, left panel). We next analyzed gene expression changes in tooth germ between these different culture conditions (Fig. 6g). qRT-PCR showed that dental epithelial cell differentiation marker genes were downregulated in the 37°C_CPA group, compared with the 37°C_DMSO group (Fig. 6g). While there was no significant difference in differentiation marker genes between the 25°C_CPA and 25°C_DMSO groups (Fig. 6g). The expression of *p21* in the 25°C_CPA group was not altered compared to that of the 25°C_DMSO group; however, in the 37°C_CPA group, it was significantly upregulated (Fig. 6g). These results indicate that the low-temperature culture can prevent CPA effects on tooth germ proliferation, apoptosis, and differentiation by suppressing DNA damage.

### Low temperature prevents CPA effects on tooth germ by temporarily halting cell cycle

**Figure 7:**
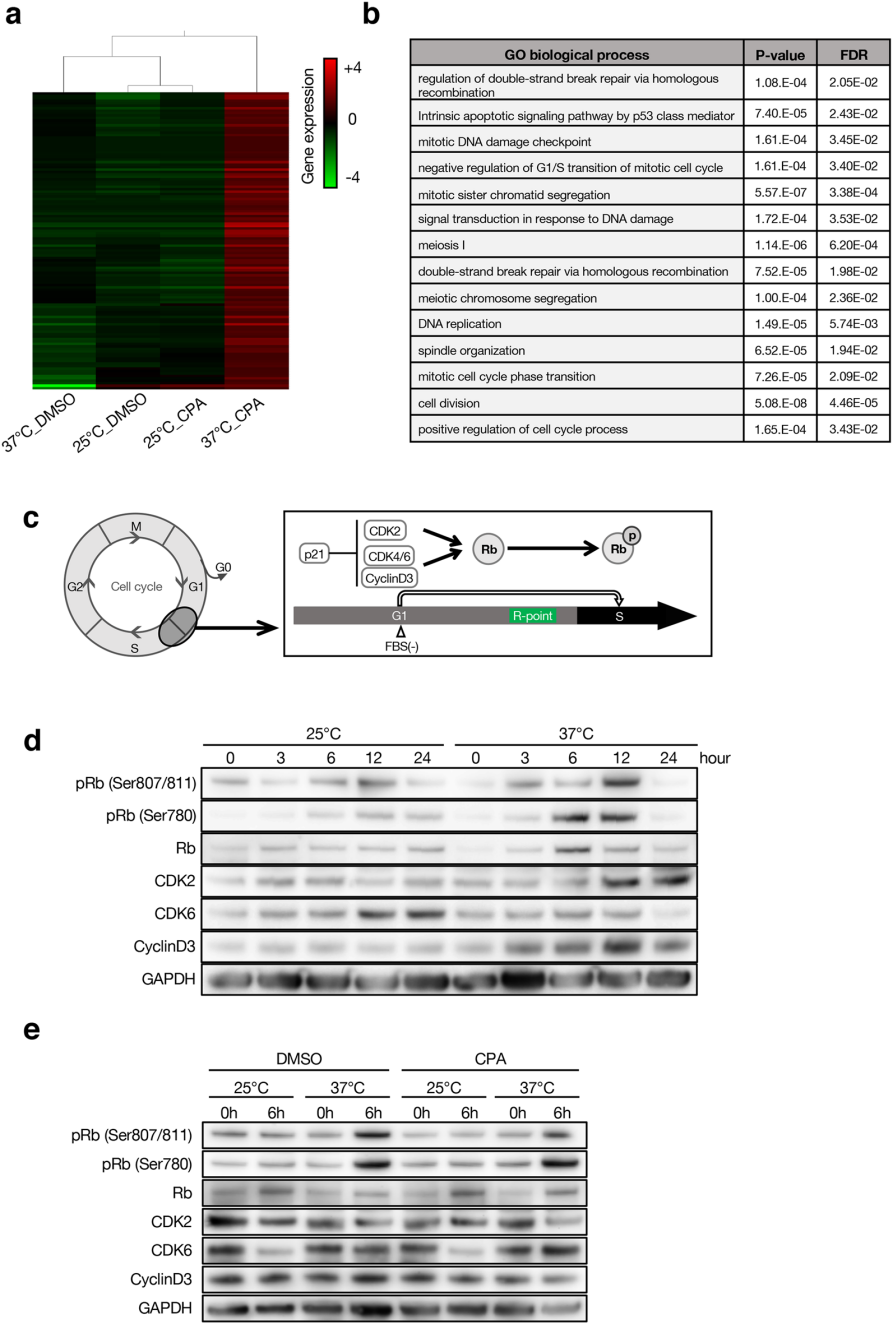
Low temperature prevents the cell cycle from G1 to M phase. (**a**) Heat map of differentially expressed genes between different organ culture systems using CAGE analysis. Datasets were obtained from > 5 independent tooth germs for each group. (**b**) Selected gene ontology term enriched in the 37°C_CPA group. (**c**) Schematic cartoon of cell cycle and the regulation mechanism of G1-phase to S-phase. (**d**) Western blotting results of pRb, CDK2, CDK6, CyclinD3 and GAPDH in M3H1 cells treated with DMSO at 25 °C or 37 °C with indicated time points. The cropped blots are presented in the figure and uncropped blots are presented in Supplementary Fig. S5a. (**e**) Western blotting results of pRb, CDK2, CDK6, CyclinD3 and GAPDH in M3H1 cells treated with DMSO or CPA at 25 °C or 37 °C with indicated time points. The cropped blots are presented in the figure and uncropped blots are presented in Supplementary Fig. S5b.

To explore the molecular mechanism of the CPA effects on tooth germ at different temperatures, we performed CAGE analysis. Cluster analysis indicated that low temperature culture can inhibit the expression level of gene clusters that were increased by CPA treatment at 37 °C (Fig. 7a). In addition, gene ontology (GO) analysis indicated that these genes were highly involved in apoptosis, DNA damage, DNA repair, and G1/S cell cycle checkpoint (Fig. 7b; Supplementary Table S1). Therefore, we focused on cell-cycle related proteins in the G1 to S phases. Rb, a cell cycle-related protein, is phosphorylated by CDK and Cyclin and plays an important role in driving the cell cycle from G1 to S phases (Fig. 7c). To examine the effect of different culturing temperatures on cell cycle, M3H1 cells were aligned to G1 phase by serum (FBS) starvation, then changed to FBS-supplemented culture medium and cultured at 25 °C and 37 °C. The expression of cell cycle molecule, pRb (Ser807/811), pRb (Ser780), Rb, CDK2, CDK6, and CyclinD3 were examined by western blotting. After adding FBS for 6–12 h, the expression levels of pRb (Ser807/811) and pRb (Ser780) in the 37 °C group were higher than those in the 25 °C group (Fig. 7d, Supplementary Fig. S6a). Moreover, the expressions of CDK2 and CyclinD3, which promote the phosphorylation of Rb, were also significantly increased in the 37 °C group (Fig. 7d). We observed differences in the expression of cell cycle related proteins after 6 h of incubation at different temperatures (Fig. 7d); therefore, we examined the CPA treatment effect at 0 and 6 h after CPA or DMSO treatment. Phosphorylation of Rb occurred in the 37 °C group, while phosphorylation was suppressed in the 25 °C group with or without CPA treatment (Fig. 7e, Supplementary Fig. S6b). These results show that at a low temperature, cells could not pass through the R point of the cell cycle and were consequently stagnated in the G1 phase.

## Discussion

In this study, we clarified the effect of CPA on tooth development using an ex vivo organ culture system. CPA treatment on tooth germ inhibited the differentiation ability and proliferation of dental cells and induced their apoptosis. These results were mirrored in the in vitro experiments using M3H1 cells. We also demonstrated that organ damage by CPA treatment could be suppressed under cold temperature.

CPA inhibited the growth of tooth germ, and the severity of CPA-mediated damage on tooth germ varied depending on the developmental stage of tooth germ when CPA was administered. These results are consistent with a previous study that analyzed the effects of intraperitoneal injection of CPA on the teeth of mice fetuses using micro-CT^3^. The rate of dental disturbances in survivors of childhood cancer is as high as 55.6%^23^, and the incidence is highest and more severe when the anticancer drug is administered at a younger age^23,34^. Thus, the dentition, type, and severity of CPA-mediated dental dysplasia correlate with the individual developmental stages of the teeth at the time of anticancer drug administration. Most studies that examine the effect of CPA on organ development employed administration of anticancer drugs to pregnant mice and observed the effects in the fetuses^2^. This would be a limitation of the study because it is necessary to consider the transfer processes of anticancer drugs from the pregnant mother to the fetus. In this study, we employed the organ culture method and succeeded in observing the direct effect of the anticancer drug on tooth germ formation in real time. CPA was added to the culture medium based on its maximum blood concentration when administered to humans^35^. As a result, we could observe the inhibition of growth of tooth germs which correlated with the stage of tooth development, similar to that observed when anticancer drugs are administered to humans. These results suggest that the organ culture method reproduces time-specific anticancer drug side effects depending on developmental stages. CPA is converted to 4-HC in the liver and passes through the bloodstream^33^. In this study, we confirmed that CPA has a sufficient inhibitory effect on organogenesis in a concentration-dependent manner and was used as a model for anticancer drugs. This result may indicate the existence of a mechanism by which activation of CPA occurs in the organ culture system. Further analysis is needed to elucidate this metabolism. We further confirmed that the low temperature can suppress the inhibition of organogenesis by another alkylating agent melphalan, which is not metabolized in the liver. These results indicate that the side effects of alkylating drugs on organ development can be avoided by maintaining the target organ under low temperature condition.

We further demonstrated that CPA-mediated DNA damage was reduced in low temperature. γ-H2AX is used for the measurement of DNA damage and several anticancer agents induces γ-H2AX resulting in cellular apoptosis^36^. This induction of DNA damage was also observed in the treatment of CPA in mice as well^37^. These facts suggest that the low temperature condition suppressed CPA-mediated DNA damage. Cell dynamics, such as metabolism and proliferation activity changes depending on the extracellular environment, and one of the major factors that influence cellular dynamics is temperature^38,39^. A previous study using V79 cells derived from Chinese hamster lung has reported that the cell cycle slows down in lower temperatures from 37 to 25 °C, and cell division almost stops at 25 °C^40^. The extension of cell cycle at a low temperature does not only result from the arrest of the current state, but also from limited phase shift^41^. In fibroblasts, the transition from S phase to G1 phase was observed at a low temperature, and eventually almost all cells stopped at G1 phase, and this phenomenon was used for cell synchronization^40^. Furthermore, we found that the cell cycle was extended in M3H1 cells cultured at a low temperature due to the inhibition of Rb protein phosphorylation, which is considered to be the most important factor in G1/S phase transition^42^. CPA-mediated growth inhibition could be avoided by keeping the tooth germ at 25 °C for the period of CPA treatment. Interestingly, similar results were obtained in hair organoid formation. These results indicate that the cell cycle was fixed in the G1 phase by low-temperature stimulation, and DNA replication is limited, suggesting that the DNA cross-linking effect of CPA in the S phase is suppressed. While this suppression of side effects might result from other mechanisms such as the changes in intracellular uptake of drugs, which may be reduced in low temperature. Further analysis on drug uptake would be necessary for the next study.

**Figure 8:**
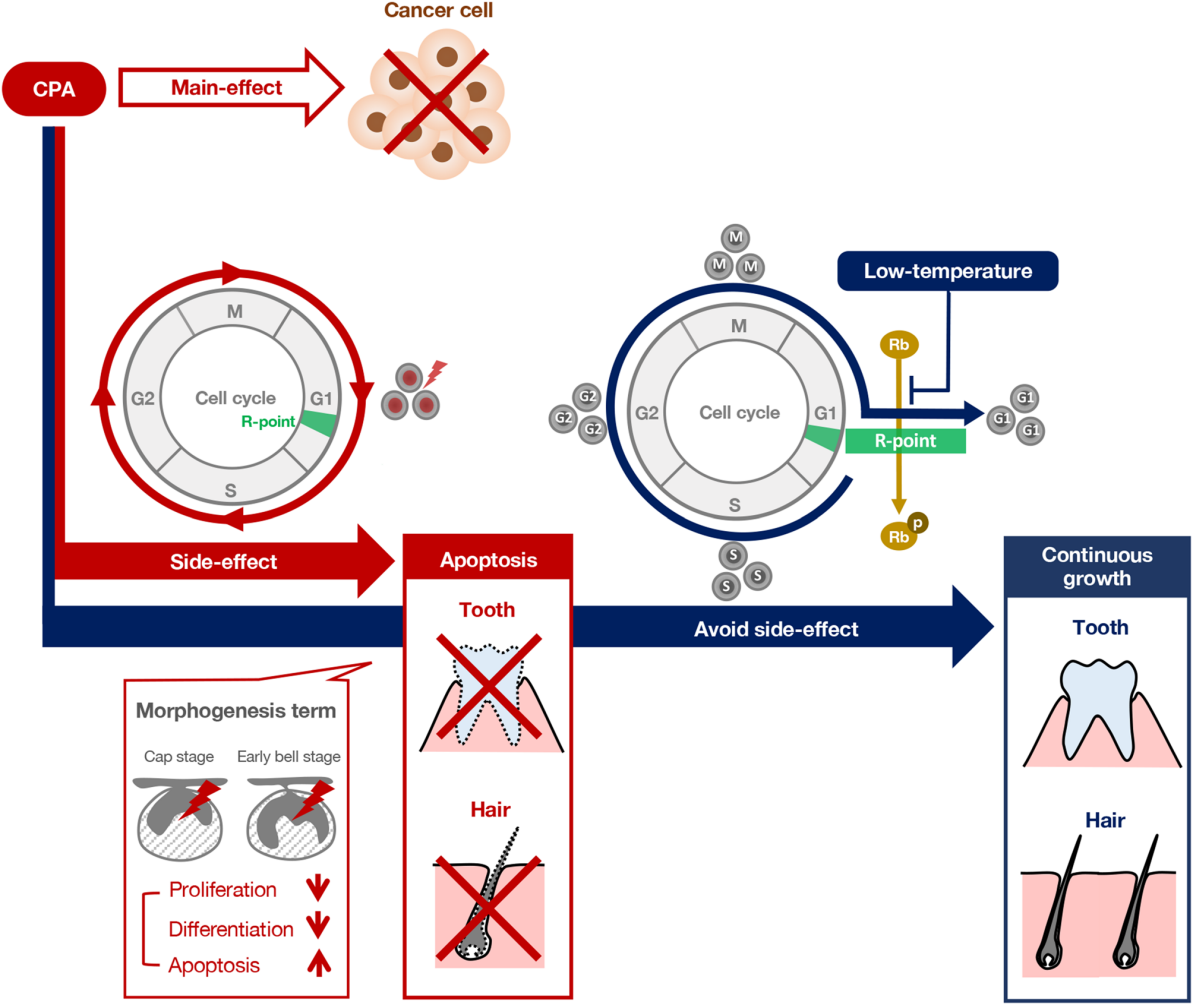
Summary diagram on the effects of CPA and a potential benefits of the low-temperature approach. Graphical summary of CPA interference on tooth germ development and the low-temperature approach to avoid side effects.

Low temperature arrests the cell cycle; hence, low temperature might prevent the side effects of anticancer drugs on organ development (Fig. 8). Furthermore, our organ culture model could be used to examine the direct effect of temperature regulation in a blood flow free organ culture environment. This fact indicates that not only vasoconstriction, but also temperature-based regulation has important roles in cryotherapy. However, since the cell cycle is arrested in all cells cultured in low temperature, there is a possibility that cooling the primary site of cancer may interfere with the main effect of anticancer drugs. Therefore, establishment of a localized and efficient cooling method is necessary to avoid the specific side effects of anticancer drugs, which will be a challenge in the future.

This study proposes a new examination model of anticancer drug side effects using an organ culture method, which is different from the conventional in vivo methods. This approach will simplify the analysis of the effects on organs in multi-chemotherapy, which is mainly used in clinical practice. Although our ex vivo experiments provide an efficient platform for drug screening, further experiments are required using in vivo models. Furthermore, we suggest that the side effects of anticancer drugs on tooth development may be avoided by maintaining the internal milieu under a low temperature.

## Methods

### Organ culture and anticancer drug treatments

All animal experiments were approved by the ethics committee of the Kyushu University Animal Experiment Center (protocol number; A20-281-0). All methods were carried out in accordance with relevant guidelines and regulations, and the study was performed in accordance with ARRIVE (Animal Research: Reporting of In Vivo experiments) guidelines. Pregnant mice were euthanized by anesthesia and the embryos were dissected immediately. Tooth germs of the mandibular first molars were dissected from mice embryos at various gestational ages (E14.5, E16.5, and E18.5). Mice whisker follicles were dissected at E13.5 then seeded into cell culture inserts (BD Falcon, BD Biosciences, Franklin Lakes, NJ, USA) and cultured using an air–liquid interface culture technique in Dulbecco’s modified Eagle’s medium (DMEM)/F-12, supplemented with 20% fetal bovine serum (FBS) (Gibco/Life Technologies, Waltham, MS, USA), 180 μg/mL ascorbic acid (Sigma, St. Louis, MS, USA), 2 mM l-glutamine (Gibco/Life Technologies, Waltham, MS, USA), and 1% penicillin/streptomycin (Gibco/Life Technologies, Waltham, MS, USA) at 37 °C in a humidified atmosphere of 5% CO_2_, as described previously^5,43^. After placing the tooth germs on inserts, different concentrations of CPA (Sigma, St. Louis, MS, USA), 1 μM of 4-HC (Toronto Research Chemicals, Inc.) or 10 μM of melphalan (Sigma, St. Louis, MS, USA) dissolved in dimethyl sulfoxide (DMSO) (Sigma, St. Louis, MS, USA) was added to a fresh organ culture medium, which was replaced every two days. The appropriate concentration of 4-HC and melphalan were selected based on previous studies^43–45^. For the control groups of tooth germ, the same volume of DMSO was added into culture medium. To assess the size of the cultured tooth germ, E14.5, E16.5, and E18.5 tooth germs were cultured for 7 days, and images were acquired under the microscope IX71 (Olympus, Tokyo, Japan) daily for record-keeping. The survival rate of cultured tooth germs was determined from morphogenesis and organ development. Tooth germs that form the cusp structure were considered as survived.

### Immunohistochemistry

For whole-mount immunolabeling, samples were fixed with 4% paraformaldehyde (Wako, Tokyo, Japan) for 15 min at room temperature, then treated with 2% Triton X-100 (Sigma, St. Louis, MS, USA) and 10% Powerblock (BioGenex, Fremont, CA, USA) in phosphate-buffered saline (PBS) for 30 min at room temperature to prevent nonspecific antigen binding. Primary antibodies were applied for 3 h followed by species-specific secondary antibodies for 2 h at room temperature with gentle shaking. Frozen sections were prepared by freezing tooth germs at − 80 °C in optimal cutting temperature (OCT) compound (Sakura, Tokyo, Japan) and slicing them at a thickness of 10 µm in LEICA CM 1860 (Leica Biosystems, Wetzlar, Germany). For immunolabeling of frozen sections and M3H1 cells, sample labeling was performed using the primary and secondary antibodies listed in Supplementary Table S2. The nuclei, tissues and cells were mounted with Vectashield mounting medium containing DAPI (Vector Laboratories, Burlingame, CA, USA). Images of cell sections were captured with a C2 confocal microscope (Nikon, Tokyo, Japan) and analyzed using NIS-Elements AR software v4.00 (Nikon, Tokyo, Japan). Images of organ samples were taken on a Zeiss LSM700 confocal laser scanning microscope (Carl Zeiss, Oberkochen, Germany) and measurement or quantification of the axial length was performed using Image J (Image J 1.51s, NIH, Bethesda, MA, USA).

### Terminal deoxynucleotidyl transferase dUTP nick end labeling (TUNEL) assay

Samples were washed with PBS, fixed with 4% paraformaldehyde for 15 min at room temperature, and washed again with PBS several times. Permeabilization Buffer (Takara Bio Inc, Tokyo, Japan) was applied on ice for 5 min and washed with PBS several times. Then, 50 μL of pre-cooled labeling reaction mixture (consisting of TdT Enzyme 5 μL + Labeling Safe Buffer 45 μL) (Takara Bio Inc, Tokyo, Japan) was added to incubate it in a 37 °C humidified chamber for 90 min and washed with PBS. Images of the cell sections were captured with a C2 confocal microscope (Nikon, Tokyo, Japan) and analyzed using NIS-Elements AR software v4.00 (Nikon, Tokyo, Japan). Images of organ samples were acquired on a Zeiss LSM700.

### Cell culture and anticancer drug treatments

 Mouse-derived dental epithelial cell line M3H1^46,47^ was maintained in Ca^2+^-free medium (Gibco/Life Technologies, Waltham, MS, USA) supplemented with 3.0 mM CaCl_2_ (Fluka Analytical, Buch, Swizerland), 1% l-glutamine (Gibco/Life Technologies, Waltham, MS, USA), 1% sodium pyruvate (Gibco/Life Technologies, Waltham, MS, USA), 1% penicillin/streptomycin (Gibco/Life Technologies, Waltham, MS, USA), and 10% Ca^2+^-free FBS (Gibco/Life Technologies, Waltham, MS, USA) at 37 °C in a humidified incubator in an atmosphere containing 5% CO_2_. For treatments, M3H1 cells were cultured in 12-well plates at a density of 2 × 10^5^ cells/well in low-Ca^2+^ DMEM. Cells were treated with different concentrations of CPA dissolved in DMSO.

### 3-(4,5-Dimethylthiazol-2-yl)-2,5-diphenylterazolium bromide (MTT) assay

M3H1 cells were seeded into 96-well plates at 0.2 × 10^4^ cells/well and treated with DMSO or CPA. Proliferation activity was measured after culturing for 1, 3, 5, and 7 days using a Cell Counting Kit (CCK)-8 (Dojindo Laboratories, Kumamoto, Japan), according to the manufacturer’s protocol. In brief, 10 µL of the CCK-8 solution was added to each well and incubated at 37 °C for 1 h. Then, the absorbance at 450 nm was measured using an iMark microplate reader (Bio-Rad, Hercules, CA, USA). The relative survival rate of cell growth was calculated, and all experiments were conducted using five wells per experiment and repeated at least three times.

### RNA isolation and real-time quantitative reverse transcription polymerase chain reaction (qRT-PCR) analysis

Total RNA was isolated from cultured cells and organ tooth germs after treatment with CPA or DMSO for 7 or 10 days, using TRIzol reagent (Life Technologies, Waltham, MS, USA) and then purified using RNeasy Mini kit (Qiagen, Venlo, Netherlands). cDNA was synthesized using SuperScript III reverse transcriptase reagent (Life Technologies, Waltham, MS, USA). The specific forward and reverse primers that were used for qRT-PCR are listed in Supplementary Table S3. Expression of each gene normalized to that of *Gapdh*. qRT-PCR was performed using iQ SYBR Green Supermix (Bio-Rad, Hercules, CA, USA) with a CFX Connect Real-Time PCR detection system (Bio-Rad, Hercules, CA, USA).

### Low-temperature culture method of organ culture

Tooth germs were cultured in the conditions previously described under various incubation temperatures. For the low-temperature system, tooth germs were cultured in an incubator at 25 °C in a humidified atmosphere of 5% CO_2_ for 7 days and treated using DMSO, 0.25 mM CPA, 1 μM 4-HC or 10 μM melphalan. For the rescue experiment, tooth germs were cultured in a medium supplemented with 0.25 mM CPA, 1 μM 4-HC or 10 μM melphalan at 25 °C for 3 days. Then, we changed to a fresh medium, only by adding DMSO, and tooth germs were cultured for an additional 4 days. Images were acquired under the microscope IX71 (Olympus, Tokyo, Japan) daily for record-keeping.

### CAGE analysis

Total RNA was isolated from cultured organs E14.5 tooth germs after adding 2.5 mM CPA or DMSO reagent for 3 days at 25 °C or 37 °C using TRIZOL reagent (Life Technologies, Waltham, MS, USA), and purified with the RNeasy Mini kit (Qiagen, Venlo, Netherlands), according to standard protocol. Bioanalyzer (Agilent, Santa Clara, CA, USA) was used to verify the quality of RNA and all samples had an RNA integrity number > 8.5. Then, CAGE analysis was performed by DNAFORM (Yokohama, Japan). The CAGE tag sequence was aligned to the reference genome (mm9), and mapped reads were subsequently converted into a CAGE defined transcription start site using SAMtools of the MOIRAI pipeline, as previously described^48^.

### Clustering analysis and gene ontology analysis

Clustering analysis was performed using Subio platform (Subio, Tokyo, Japan). Greater than twofold differentially expressed genes between the groups were used for the analysis (Supplementary Table S4). Gene ontology analysis was performed using the Gene Ontology Consortium (http://geneontology.org).

### Western blotting

Cells were lysed in CelLytic M (Sigma-Aldrich, St. Louis, MS, USA) buffer containing 1% protease inhibitor mixture (Sigma-Aldrich, St. Louis, MS, USA) and 1 mM phenylmethylsulfonyl fluoride (Sigma-Aldrich, St. Louis, MS, USA). Cell lysates were collected, and protein concentration was measured by Pierce BCA Protein Assay kit (Thermo Fisher Scientific, Waltham, MS, USA). Then, protein was denatured at 70 °C for 10 min and 10 μg of protein was ran on a 4–12% SDS–polyacrylamide gel (NuPAGE, Invitrogen, Waltham, MS, USA). Following electrophoresis, the proteins were transferred to a PVDF membrane (Life Technologies, Waltham, MS, USA), analyzed by western blotting, and incubated with the antibodies listed in Supplementary Table S2. The membrane was developed using ECL Plus reagent (Thermo Fisher Scientific, Waltham, MS, USA). Then, signals were detected using an ECL kit (Amersham Biosciences, Amersham, UK) and visualized with an ImageQuant LAS 4000 system (GE Healthcare, Chicago, IL, USA).

### Statistical analysis

All experiments were repeated at least three times to confirm the reproducibility. Statistical significance was determined using a two-tailed unpaired Student's *t* test and multiple analyses were performed using two-way ANOVA, with Prism version 6 (GraphPad Software, La Jolla, CA, USA). A *p* value < 0.05 was considered statistically significant.

## Supplementary Information

Supplementary Information.

## Data Availability

The CAGE datasets generated for this study can be found in the NCBI GEO: GSE182499. All other the data generated in this study are reported in this article (and in its Supplementary Information).

## References

[1] Koppang HS (1978). Histomorphologic investigations of dentinogenesis in incisors of offspring of cyclophosphamide-treated pregnant rats. Scand. J. Dent. Res..

[2] Kawakami T, Nakamura Y, Karibe H (2015). Cyclophosphamide-induced morphological changes in dental root development of ICR mice. PLoS One.

[3] Nakatsugawa K (2019). Stage- and tissue-specific effect of cyclophosphamide during tooth development. Eur. J. Orthod..

[4] Karagözoğlu S, Filiz Ulusoy M (2005). Chemotherapy: The effect of oral cryotherapy on the development of mucositis. J. Clin. Nurs..

[5] Han X (2020). Mouse embryonic tooth germ dissection and ex vivo culture protocol. Bio-Protoc..

[6] Houghton PJ, Kurmasheva RT (2019). Challenges and opportunities for childhood cancer drug development. Pharmacol. Rev..

[7] Gebauer J (2018). Late effects following childhood cancer treatment: A special challenge for transition medicine. Internist (Berl).

[8] Siegel RL, Miller KD, Jemal A (2017). Cancer statistics, 2017. CA Cancer J. Clin..

[9] Malard F, Mohty M (2020). Acute lymphoblastic leukaemia. Lancet.

[10] Malhotra V, Perry MC (2003). Classical chemotherapy: Mechanisms, toxicities and the therapeutic window. Cancer Biol. Ther..

[11] de Jonge ME, Huitema AD, Rodenhuis S, Beijnen JH (2005). Clinical pharmacokinetics of cyclophosphamide. Clin. Pharmacokinet..

[12] Homan ER, Zendzian RP, Busey WM, Rall DP (1969). Loss of hair in experimental animals induced by cyclophosphamide 19. Nature.

[13] Paus R, Haslam IS, Sharov AA, Botchkarev VA (2013). Pathobiology of chemotherapy-induced hair loss. Lancet Oncol..

[14] van Oort S, Kramer E, de Groot JW, Visser O (2018). Taste alterations and cancer treatment. Curr. Opin. Support Palliat. Care.

[15] Delay ER (2019). Cyclophosphamide and the taste system: Effects of dose fractionation and amifostine on taste cell renewal. PLoS One.

[16] Mukherjee N, Pal Choudhuri S, Delay RJ, Delay ER (2017). Cellular mechanisms of cyclophosphamide-induced taste loss in mice. PLoS One.

[17] Nemeth O (2014). Late effects of multiagent chemotherapy on salivary secretion in children cancer survivors. J. Am. Coll. Nutr..

[18] Goho C (1993). Chemoradiation therapy: Effect on dental development. Pediatr. Dent..

[19] Krasuska-Sławińska E, Brożyna A, Dembowska-Bagińska B, Olczak-Kowalczyk D (2016). Antineoplastic chemotherapy and congenital tooth abnormalities in children and adolescents. Contemp. Oncol. (Pozn.).

[20] Balic A, Thesleff I (2015). Tissue interactions regulating tooth development and renewal. Curr. Top. Dev. Biol..

[21] Ishikawa Y, Ida-Yonemochi H, Nakakura-Ohshima K, Ohshima H (2012). The relationship between cell proliferation and differentiation and mapping of putative dental pulp stem/progenitor cells during mouse molar development by chasing BrdU-labeling. Cell Tissue Res..

[22] Nakasone N, Yoshie H, Ohshima H (2006). The relationship between the termination of cell proliferation and expression of heat-shock protein-25 in the rat developing tooth germ. Eur. J. Oral Sci..

[23] Kang CM (2018). Clinical risk factors influencing dental developmental disturbances in childhood cancer survivors. Cancer Res. Treat..

[24] Riley P, McCabe MG, Glenny AM (2016). Oral cryotherapy for preventing oral mucositis in patients receiving cancer treatment. JAMA Oncol..

[25] Peyton L, Fischer-Cartlidge E (2019). Extremity cooling: A synthesis of cryotherapy interventions to reduce peripheral neuropathy and nail changes from taxane-based chemotherapy. Clin. J. Oncol. Nurs..

[26] Cascinu S, Fedeli A, Fedeli SL, Catalano G (1994). Oral cooling (cryotherapy), an effective treatment for the prevention of 5-fluorouracil-induced stomatitis. Eur. J. Cancer B Oral Oncol..

[27] Osaki A (2020). Drinking ice-cold water reduces the severity of anticancer drug-induced taste dysfunction in mice. Int. J. Mol. Sci..

[28] He B (2019). Identification of the novel tooth-specific transcription factor AmeloD. J. Dent. Res..

[29] Han X (2018). The transcription factor NKX2-3 mediates p21 expression and ectodysplasin-A signaling in the enamel knot for cusp formation in tooth development. J. Biol. Chem..

[30] Yoshizaki K, Fukumoto S, Bikle DD, Oda Y (2020). Transcriptional regulation of dental epithelial cell fate. Int. J. Mol. Sci..

[31] Huang F (2015). Expression profile of critical genes involved in FGF signaling pathway in the developing human primary dentition. Histochem. Cell Biol..

[32] Zhang Y (2000). A new function of BMP4: Dual role for BMP4 in regulation of Sonic hedgehog expression in the mouse tooth germ. Development.

[33] Boddy AV, Yule SM (2000). Metabolism and pharmacokinetics of oxazaphosphorines. Clin. Pharmacokinet..

[34] Effinger KE (2014). Oral and dental late effects in survivors of childhood cancer: A Children's Oncology Group report. Support Care Cancer.

[35] Ramirez DA, Collins KP, Aradi AE, Conger KA, Gustafson DL (2019). Kinetics of cyclophosphamide metabolism in humans, dogs, cats, and mice and relationship to cytotoxic activity and pharmacokinetics. Drug Metab. Dispos..

[36] Kuo LJ, Yang LX (2008). Gamma-H2AX—a novel biomarker for DNA double-strand breaks. In Vivo.

[37] Chen Q (2021). Epigallocatechin gallate and theaflavins independently alleviate cyclophosphamide-induced ovarian damage by inhibiting the overactivation of primordial follicles and follicular atresia. Phytomedicine.

[38] Zhang Q, Austin RH (2012). Applications of microfluidics in stem cell biology. BioNanoScience.

[39] Mäki AJ (2018). A portable microscale cell culture system with indirect temperature control. SLAS Technol..

[40] Nelson RJ, Kruuv J (1972). Survival of synchronized mammalian cells following exposure to cold. Exp. Cell Res..

[41] Shapiro IM, Lubennikova EI (1968). Population kinetics of cells in tissue culture incubated at low temperature. Exp. Cell Res..

[42] Giacinti C, Giordano A (2006). RB and cell cycle progression. Oncogene.

[43] Liu R (2020). Melphalan induces cardiotoxicity through oxidative stress in cardiomyocytes derived from human induced pluripotent stem cells. Stem Cell Res. Ther..

[44] Gajek A (2020). Chemical modification of melphalan as a key to improving treatment of haematological malignancies. Sci. Rep..

[45] Strauss G (2008). 4-hydroperoxy-cyclophosphamide mediates caspase-independent T-cell apoptosis involving oxidative stress-induced nuclear relocation of mitochondrial apoptogenic factors AIF and EndoG. Cell Death Differ..

[46] Arai C (2017). Nephronectin plays critical roles in Sox2 expression and proliferation in dental epithelial stem cells via EGF-like repeat domains. Sci. Rep..

[47] Yoshizaki K (2017). Mediator 1 contributes to enamel mineralization as a coactivator for Notch1 signaling and stimulates transcription of the alkaline phosphatase gene. J. Biol. Chem..

[48] Funada K (2020). microRNA-875-5p plays critical role for mesenchymal condensation in epithelial-mesenchymal interaction during tooth development. Sci. Rep..

