# Supplementary material for: An ex vivo organ culture screening model revealed that low temperature conditions prevent side effects of anticancer drugs
Source: Sci Rep. 2022 Feb 23;12:3093. doi: 10.1038/s41598-022-06945-7 (PMC8866511; doi:10.1038/s41598-022-06945-7)
Supplement: Supplementary file 1 — Supplementary Information. [file 41598_2022_6945_MOESM1_ESM.pdf]

Supplemental information

An ex vivo organ culture screening model revealed that low temperature conditions prevent side effects of anticancer drugs

Tian Tian, Kanako Miyazaki, Yuta Chiba, Keita Funada, Tomomi Yuta, Kanji Mizuta, Yao Fu, Jumpei Kawahara, Xue Han, Yuna Ando, Ami Funada, Aya Yamada, Tsutomu Iwamoto, Seiji Nakamura, Ichiro Takahashi, Satoshi Fukumoto, Keigo Yoshizaki

Supplementary Figure 1. Morphogenesis of tooth germs supplemented with CPA.

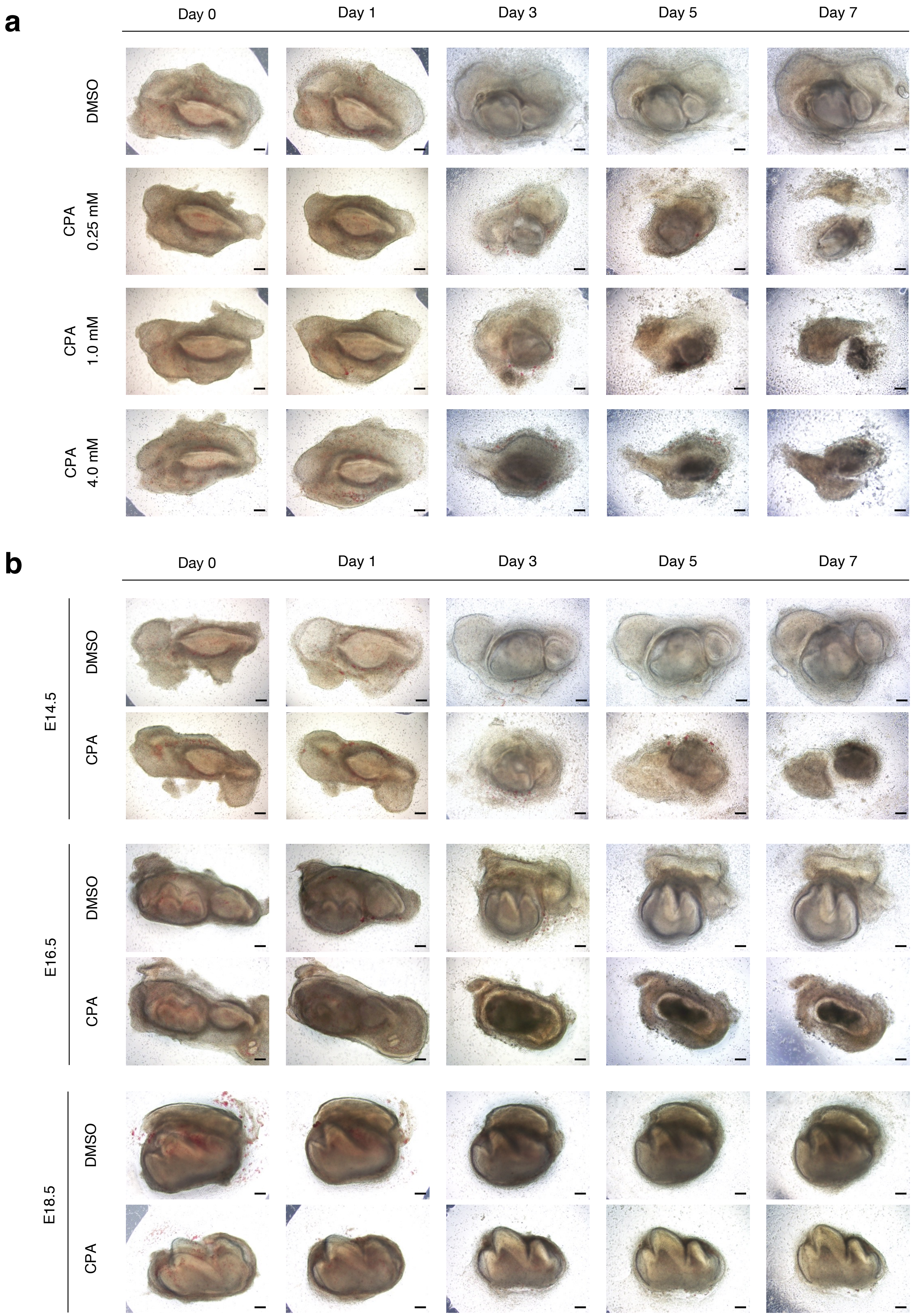

(a) Morphology of E14.5 tooth germs cultured for 0, 1, 3, 5 and 7-day in organ culture system, treated with DMSO or CPA (0.25 mM, 1.0 mM, 4.0 mM) (n = 10).
(b) Morphology of different developmental stage of tooth germs cultured for 0, 1, 3, 5 and 7-day in organ culture system, treated with DMSO or 0.25 mM of CPA (n = 8).
Scale bars, 200 μm.

Supplementary Figure 2. The expression of CK14 and vimentin in CPA-treated tooth germs.

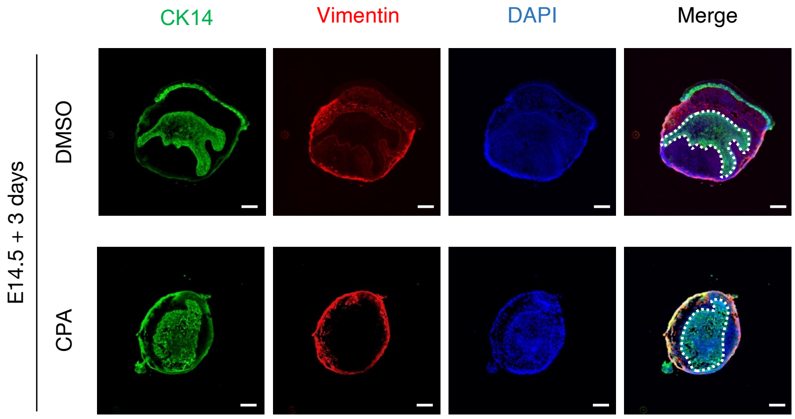

Immunofluorescence of CK14 (green) and Vimentin (red) in the section of E14.5 tooth germs cultured for three-day treated with DMSO or 0.25 mM of CPA. Nuclei were stained with DAPI (blue). Scale bars, 200 μm.

Supplementary Figure 3. The damaged tooth germs by CPA cannot be rescued by the addition of growth factors.

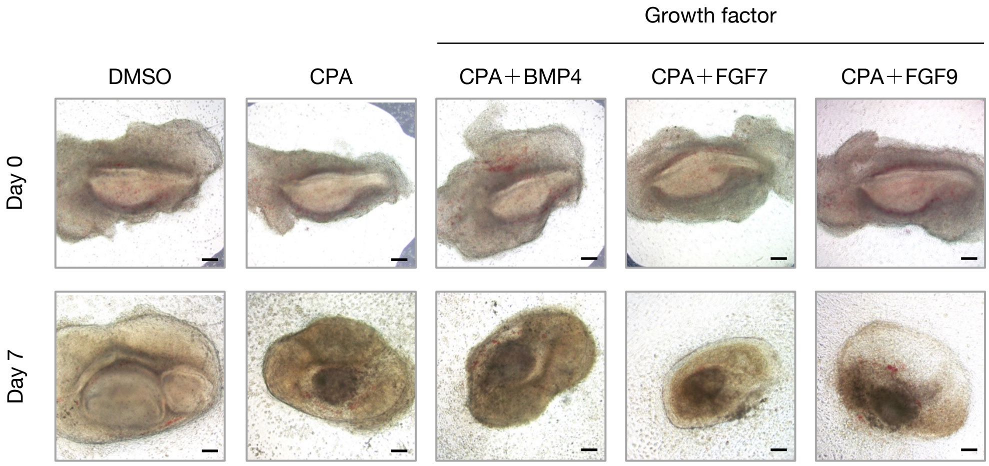

Morphology of E14.5 tooth germs cultured for seven-day treated with DMSO, 0.25 mM of CPA, or 0.25 mM of CPA with growth factor (BMP4, FGF7, FGF9) (n = 8). Scale bars, 200 μm.

Supplementary Figure 4. Low-temperature culture method decreases the 4-hydroxycyclophaophamide (4-HC)-mediated damage on the growth of culture organs.

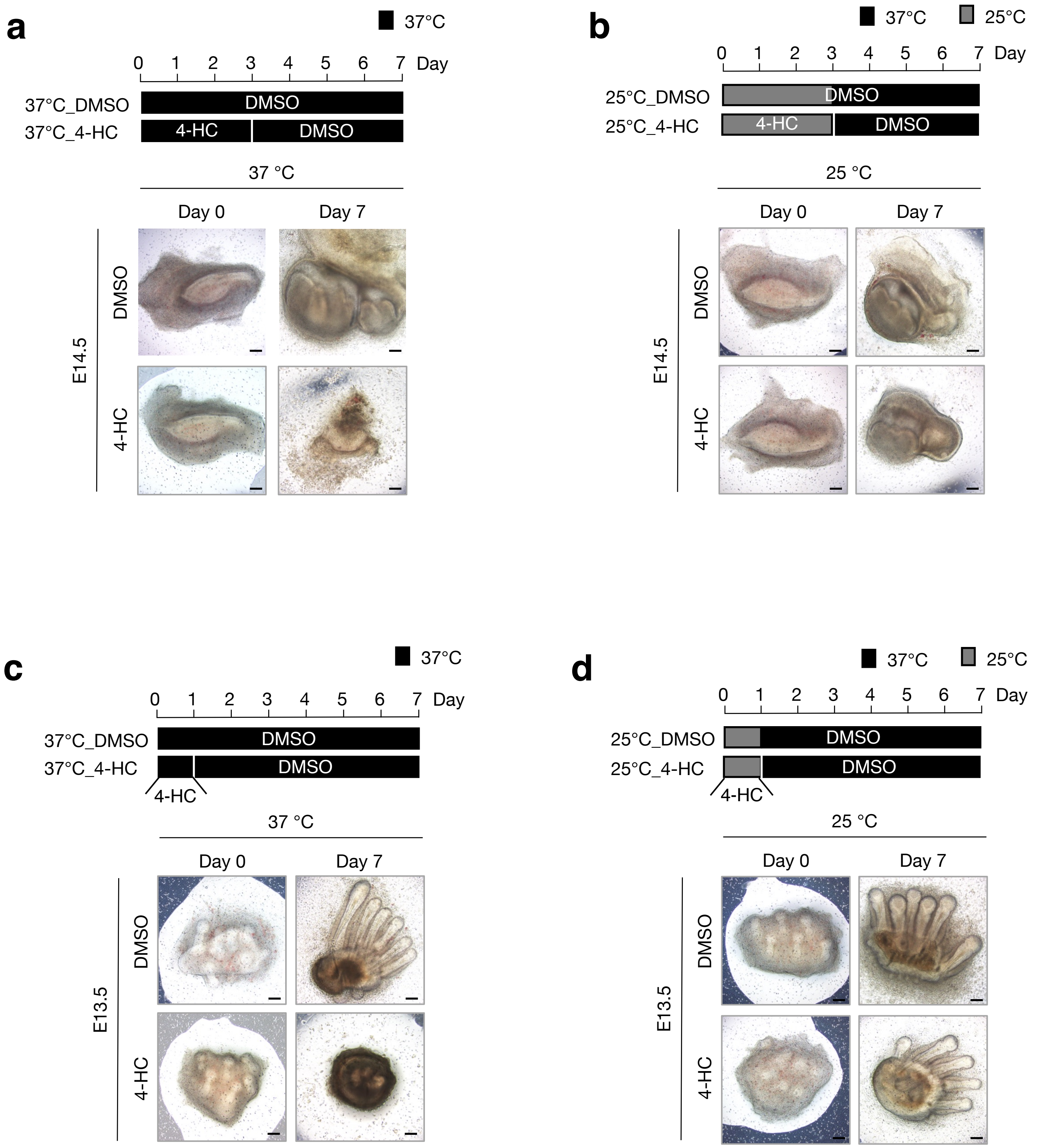

(a) Morphology of tooth germs in a conventional method of organ culture. In the first three days, tooth germs were treated with DMSO or 1μM of 4-HC (Toronto Research Chemicals, Inc.), then the culturing medium was replaced to that of without 4-HC for additional four days. The temperature was maintained at 37°C throughout incubation of tooth germs.

(b) Morphology of tooth germs in a low-temperature culture method of organ culture. Tooth germs were treated with DMSO or 1μM of 4-HC at 25°C in the first three days, then the culturing medium was replaced to that of without 4-HC for additional four days at 37°C.

(c) Organ cultured E13.5 hair tissues for 7 days with normal method.

(d) E13.5 hair tissues were organ cultured for 7 days with low-temperature method.

Scale bars, 200 μm.

Supplementary Figure 5. Low-temperature culture method decreases the melphalan-mediated damage on the growth of culture organs.

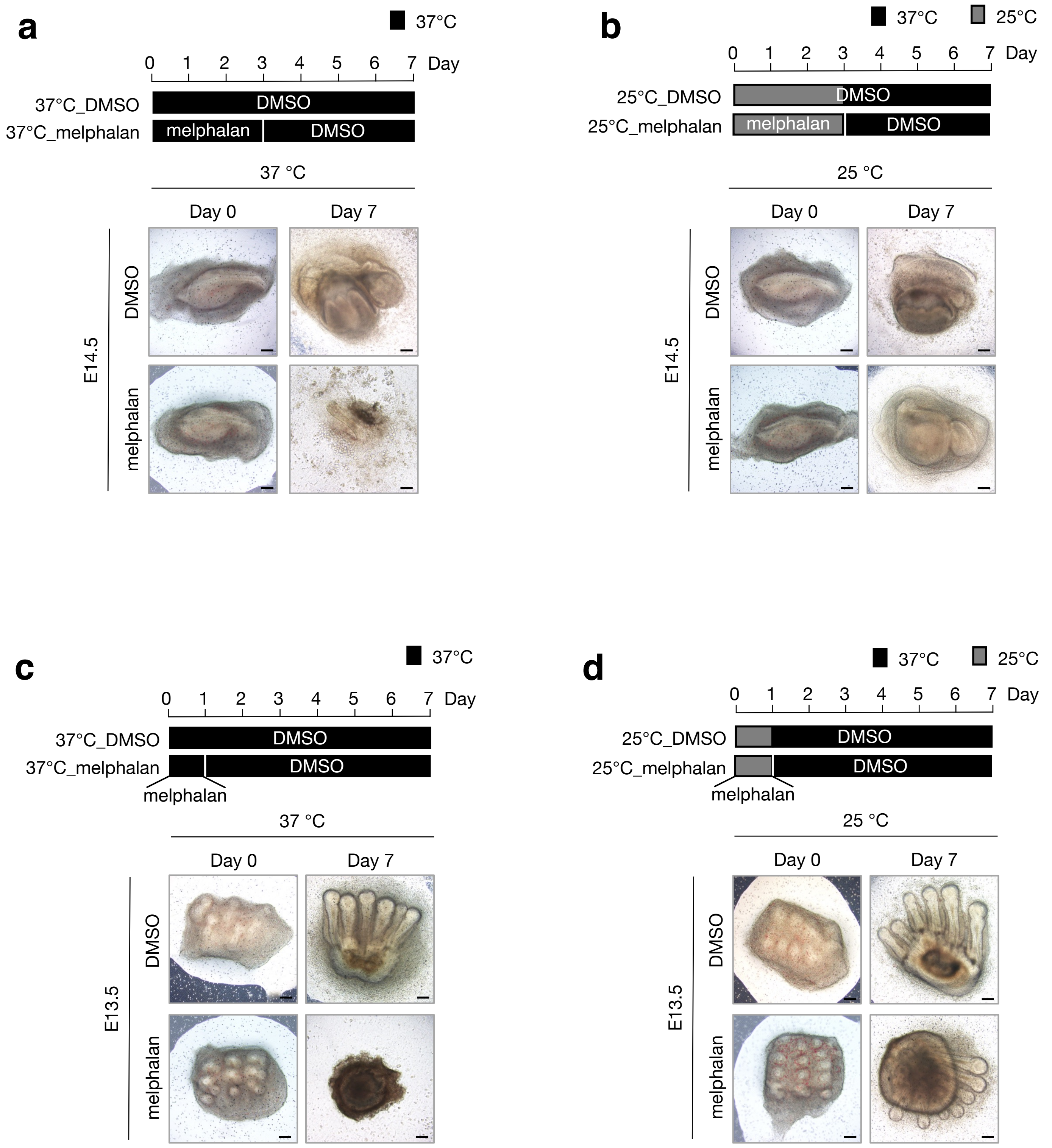

(a) Morphology of tooth germs in a conventional method of organ culture. In the first three days, tooth germs were treated with DMSO or 10 μM of melphalan (Sigma. St. Louis, MS, USA), then the culturing medium was replaced to that of without melphalan for additional four days. The temperature was maintained at 37°C throughout incubation of tooth germs.
(b) Morphology of tooth germs in a low-temperature culture method of organ culture. Tooth germs were treated with DMSO or 10 μM of melphalan at 25°C in the first three days, then the culturing medium was replaced to that of without melphalan for additional four days at 37°C.
(c) Organ cultured E13.5 hair tissues for 7 days with normal method.
(d) E13.5 hair tissues were organ cultured for 7 days with low-temperature method.
Scale bars, 200 μm.

Supplementary Figure 6. The uncropped blots presented in Fig. 7.

a

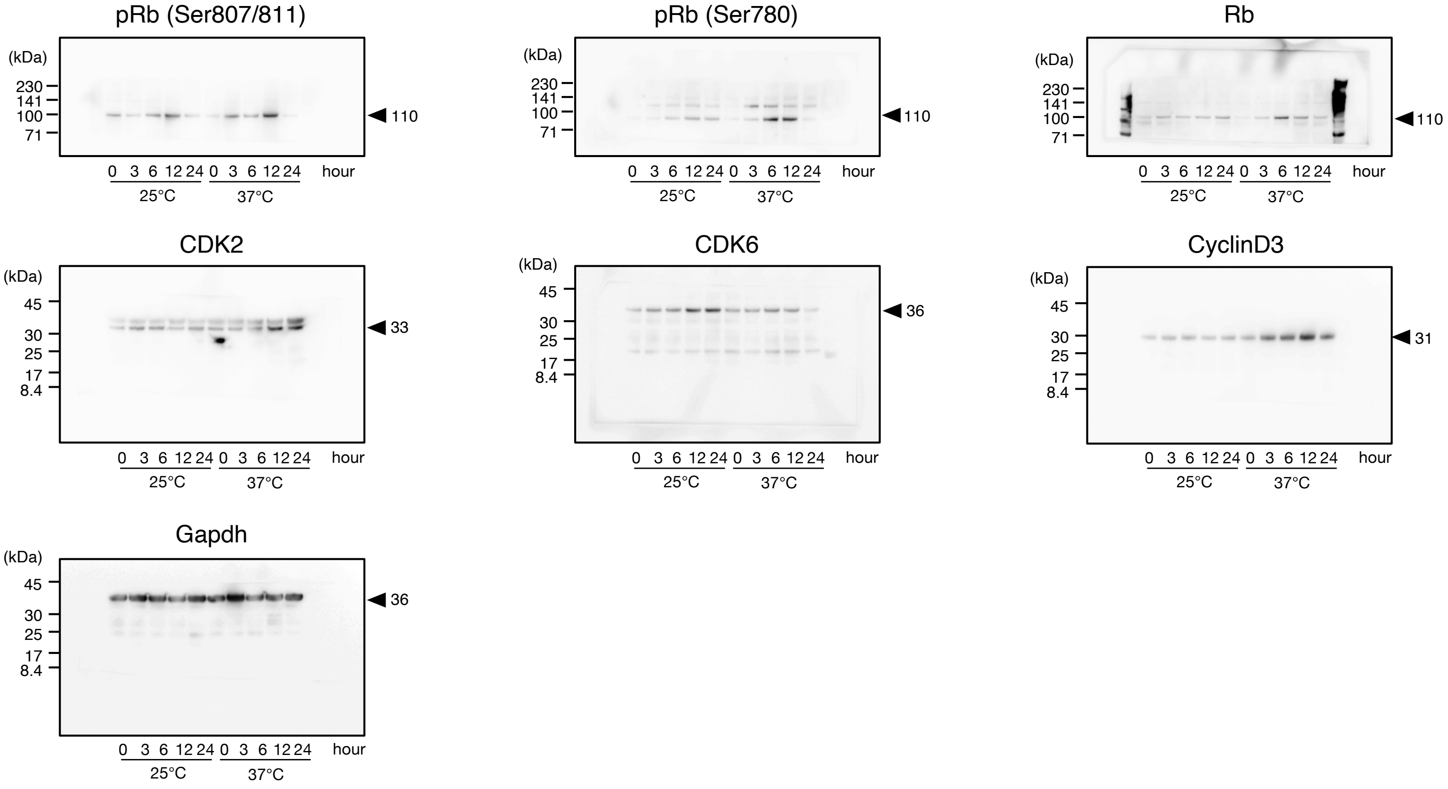

b

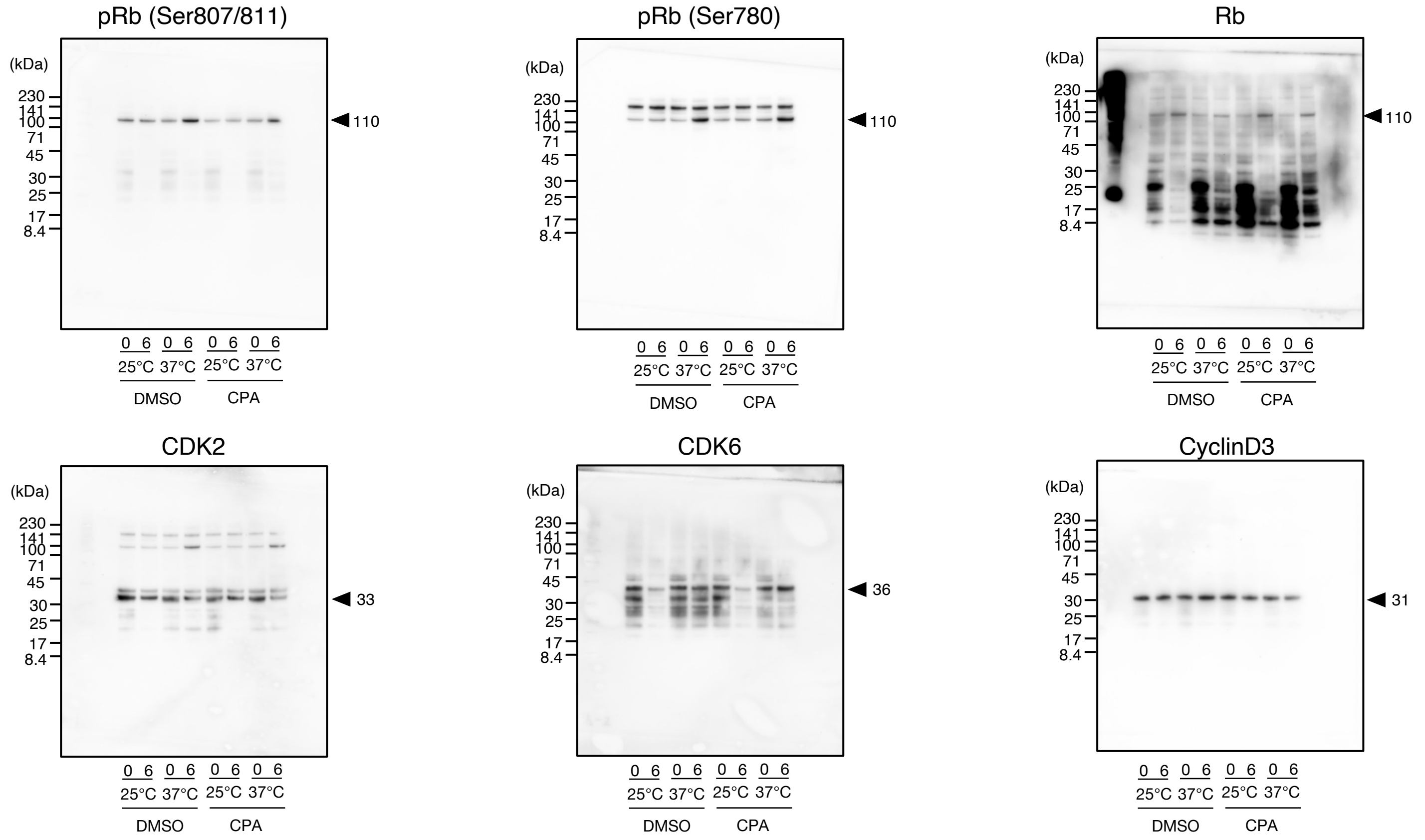

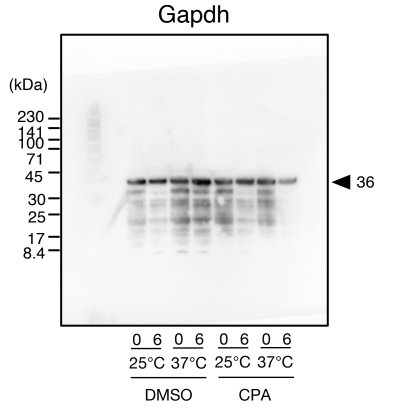

(a) Uncropped blots of anti-pRb (Ser807/811), anti-pRb (Ser780), anti-Rb, anti-CDK2, anti-CDK6, anti-CyclinD3 and anti-Gapdh related to Fig. 7d. Molecular weight was predicted with standard protein marker.

(b) Uncropped blots of anti-pRb (Ser807/811), anti-pRb (Ser780), anti-Rb, anti-CDK2, anti-CDK6, anti-CyclinD3 and anti-Gapdh related to Fig. 7e. Molecular weight was predicted with standard protein marker.

Supplementary Table 1. Full-list of gene ontology term related to Fig. 7b.

GO biological process	list of genes									
regulation of double-strand break repair via homologous recombination	Tex15	Rad51ap1	Zfp365	Rad51						
intrinsic apoptotic signaling pathway by p53 class mediator	Bbc3	Eda2r	E2f2	Aen						
mitotic DNA damage checkpoint	Fancd2	Eme1	Ccng1	Pidd1						
negative regulation of G1/S transition of mitotic cell cycle	E2f7	Plk5	Pidd1	Haspin						
mitotic sister chromatid segregation	Ncapg	Kif18b	Espl1	Spag5	Cdca5	Psrc1	Haspin			
signal transduction in response to DNA damage	E2f7	Sesn2	Brca1	Pidd1						
meiosis I	Fancd2	Tex15	Espl1	Ccne2	Eme1	Mybl1	Rad51			
double-strand break repair via homologous recombination	Rad51ap1	Brca1	Aunip	Cdc45	Rad51					
meiotic chromosome segregation	Fancd2	Tex15	Espl1	Ccne2	Eme1					
DNA replication	Rrm2	Polk	Ccne2	Eme1	Fam111a	Cdc45	Rad51			
spindle organization	Espl1	Kifc1	Kifc5b	Spag5	Aunip	Klf11				
mitotic cell cycle phase transition	Tcf19	E2f7	Ube2c	Ccne2	Ccng1	Iqgap3				
cell division	Plk5	Kif18b	Ube2c	Ccne2	Kntc1	Spag5	Cdca5	Ercc6l	Zwilch	Zfp365
	Ckap2	Ccng1	Psrc1	Kif11						
positive regulation of cell cycle process	E2f7	Ube2c	Brca1	Spag5	Cdca5	Pidd1	Psrc1	Fosl1		

Supplementary Table 2. Antibodies used in this study.

Antibody	Purpose	Dilution	Provider
**Anti-Ki67**	Whole-mount immunostaining	1:100	Cell Signaling Technology, Danvers, MS, USA
**Anti-Perlecan**	Whole-mount immunostaining	1:100	Invitrogen, Waltham, MS, USA
**Alexa 488 or Alexa 594 fluorescent dye**	Whole-mount immunostaining	1:250	Life Technologies, Waltham, MS, USA
**Anti-Ki67**	Immunostaining	1:500	Cell Signaling Technology, Danvers, MS, USA
**Anti-Perlecan**	Immunostaining	1:500	Invitrogen, Waltham, MS, USA
**Anti-p21**	Immunostaining	1:500	abcam, Cambridge, UK
**Anti-γ-H2AX**	Immunostaining	1:500	Cell Signaling Technology, Danvers, MS, USA
**Anti-Keratin 14**	Immunostaining	1:500	Covance, Princeton, NJ, USA
**Anti-Vimentin**	Immunostaining	1:500	Santa Cruz, Dallas, TX, USA
**Alexa 488 or Alexa 594 fluorescent dye**	Immunostaining	1:500	Life Technologies, Waltham, MS, USA
**Anti-pRb(Ser807/811)**	Western blotting	1:500	Cell Signaling Technology, Danvers, MS, USA
**Anti-pRb(Ser780)**	Western blotting	1:500	Cell Signaling Technology, Danvers, MS, USA
**Anti-Rb**	Western blotting	1:500	Cell Signaling Technology, Danvers, MS, USA
**Anti-CDK2**	Western blotting	1:500	Cell Signaling Technology, Danvers, MS, USA
**Anti-CDK6**	Western blotting	1:500	Cell Signaling Technology, Danvers, MS, USA
**Anti-CyclinD3**	Western blotting	1:500	Cell Signaling Technology, Danvers, MS, USA
**Anti-GAPDH**	Western blotting	1:500	Cell Signaling Technology, Danvers, MS, USA
**Horseradish peroxidase-conjugated secondary antibody**	Western blotting	1:1000	Cell Signaling Technology, Danvers, MS, USA

Supplementary Table 3. Primer sequences used in this study.

Gene	Forward	Reverse
Amelogenin	5’-ctgtctcaacagcatccc -3’	5’-caggaactggcatcattg-3’
Ameloblastin (Ambn)	5’-acaacgcatggcgtttccaa-3’	5’-accttcactgcggaaggata-3’
AmeloD	5’-actacgacgcctacactggg-3’	5’-atgaaggcaggctcgaacgg-3’
Nkx2-3	5’-tggccctgatgatgttacca-3’	5’-ggaaaactgcgtcccttcag-3’
p21	5’-tcccgactcttgacattgct-3’	5’-tccaaaatagaggggcagct-3’
**glyceraldehyde 3-phosphate dehydrogenase (*Gapdh*)**	5’-ggagcgagaccccactaacatc-3’	5’-ctcgtggttcacacccatcac-3’

Supplementary Table 4. Full-list of genes upregulated in 37°C_CPA.

	25_DMSO	25_CPA		37_DMSO	37_CPA	
Gene	raw signal	raw signal	FC	raw signal	raw signal	FC
Tcf19	181	183	1.0	227	502	2.2
H2-Eb1	7	1	0.1	13	82	6.3
Eda2r	60	55	0.9	64	442	6.9
Spag5	49	45	0.9	69	158	2.3
Rhbdl2	44	47	1.1	62	140	2.3
Adrb2	31	42	1.4	53	122	2.3
Melk	181	172	1.0	241	480	2.0
Eme1	66	74	1.1	108	235	2.2
Gm13067	52	50	1.0	38	319	8.4
Cdc42bpg	128	167	1.3	205	545	2.7
Inka2	44	40	0.9	79	428	5.4
Hrob	69	57	0.8	90	192	2.1
Gm25791	10	2	0.2	37	79	2.1
9230114K14Rik	52	66	1.3	48	189	3.9
Fam124a	81	90	1.1	35	171	4.9
Cox6b2	133	150	1.1	226	1059	4.7
Kif18b	241	242	1.0	271	684	2.5
Gm23935	2985	3170	1.1	2746	8958	3.3
Slfn9	106	110	1.0	110	284	2.6
Ncapg	388	420	1.1	545	1177	2.2
Pidd1	85	105	1.2	72	318	4.4
Kntc1	106	119	1.1	141	396	2.8
Bbc3	158	125	0.8	185	410	2.2
Ano3	14	10	0.7	4	214	53.5
Slc19a2	334	360	1.1	283	1012	3.6
Trp53inp1	227	243	1.1	209	1063	5.1
Kif11	380	352	0.9	569	1050	1.8
Ephx1	102	88	0.9	198	1029	5.2
Gm22579	10	2	0.2	37	79	2.1
Dglucy	78	70	0.9	85	353	4.2
1500009L16Rik	182	150	0.8	286	683	2.4
Mns1	35	30	0.9	35	92	2.6
Ccne2	477	428	0.9	550	1635	3.0
Neil3	185	212	1.1	233	580	2.5
Ercc6l	110	133	1.2	154	342	2.2
Gm27032	0	1	0.0	35	163	4.7
Zfp365	105	139	1.3	65	284	4.4
Kifc5b	100	105	1.1	93	295	3.2
2310008N11Rik	22	15	0.7	9	110	12.2
Mir124a-3	0	1	0.0	35	163	4.7
Btg2	431	528	1.2	795	1967	2.5
1700007K13Rik	61	76	1.2	84	532	6.3
Iqgap3	74	72	1.0	115	224	1.9
Fancd2	83	90	1.1	106	295	2.8
Slc2a9	88	98	1.1	71	214	3.0
Tex15	21	19	0.9	43	159	3.7
Exo1	152	144	0.9	126	388	3.1
Rrm2	455	454	1.0	430	1332	3.1
Gtse1	198	229	1.2	385	1302	3.4
Abcb1b	17	20	1.2	29	370	12.8
Kifc1	204	247	1.2	303	757	2.5
Brca1	173	222	1.3	203	571	2.8
Gm22620	10	2	0.2	37	79	2.1
Gm17511	3276	4628	1.4	120	5326	44.4
Bard1	205	193	0.9	257	590	2.3
Gm3776	7	4	0.6	14	103	7.4
Tnfrsf10b	301	302	1.0	277	758	2.7
Polk	224	190	0.8	338	1034	3.1
Rnf169	27	38	1.4	29	100	3.4
E2f2	35	42	1.2	44	140	3.2
Gipc2	20	8	0.4	8	90	11.3
Mtfr2	100	125	1.3	95	262	2.8
Rad51	539	588	1.1	502	1525	3.0
Sesn2	286	283	1.0	312	968	3.1
Ddias	219	213	1.0	134	912	6.8
Fanci	53	65	1.2	85	224	2.6
Plk5	4	8	2.0	18	311	17.3
Ube2c	1315	1344	1.0	2086	4095	2.0
Fosl1	57	65	1.1	85	183	2.2
Tubb4b	1678	1727	1.0	2229	5256	2.4
Esco2	254	233	0.9	361	824	2.3
Gm10073	112	156	1.4	4	223	55.8
Psrc1	228	210	0.9	348	1332	3.8
Ckap2	345	375	1.1	360	1030	2.9
Gm12981	286	283	1.0	312	968	3.1
Cenpi	76	95	1.3	71	197	2.8
Arntl2	5	14	2.8	46	213	4.6
Cdca5	156	156	1.0	214	428	2.0
Cmpk2	20	26	1.3	42	85	2.0
Gm49349	10	2	0.2	37	79	2.1
Celf5	20	30	1.5	53	339	6.4
Acaa1b	120	132	1.1	79	1030	13.0
Ccng1	1213	1222	1.0	1052	4878	4.6
Steap1	20	11	0.6	36	76	2.1
Haspin	55	50	0.9	53	154	2.9
Fam111a	151	185	1.2	252	503	2.0
Ankle1	63	69	1.1	105	231	2.2
Trim7	8	1	0.1	26	176	6.8
Dcxr	225	229	1.0	105	780	7.4
Aunip	70	44	0.6	95	251	2.6
Cdc45	362	302	0.8	522	994	1.9
Scn1b	13	11	0.8	27	75	2.8
Gm10221	50	2	0.0	21	271	12.9
Kif15	213	175	0.8	296	824	2.8
Capsl	15	9	0.6	30	91	3.0
Gm5424	231	207	0.9	323	791	2.4
Nkx2-9	0	2	0.0	0	58	0.0
Tnfrsf18	25	38	1.5	51	243	4.8
A930001C03Rik	32	30	0.9	24	186	7.8
Ass1	32	32	1.0	39	121	3.1
Rad51ap1	113	148	1.3	191	398	2.1
Shcbp1	127	139	1.1	201	429	2.1
Mgmt	268	206	0.8	156	506	3.2
Aen	727	787	1.1	665	2011	3.0
Rprm	49	43	0.9	29	105	3.6
Hmgcll1	30	52	1.7	74	192	2.6
Zwilch	152	122	0.8	100	316	3.2
Gm23455	10	2	0.2	37	79	2.1
Exoc4	322	331	1.0	372	1131	3.0
Zmat3	300	256	0.9	279	1030	3.7
Fbxo48	42	39	0.9	41	136	3.3
Svop	2	3	1.5	0	63	0.0
Lrr1	133	148	1.1	107	341	3.2
Mybl1	90	100	1.1	82	443	5.4
Ms4a10	14	10	0.7	3	122	40.7
Espl1	73	63	0.9	116	273	2.4
E2f7	169	163	1.0	259	552	2.1
Scn3b	37	36	1.0	15	89	5.9
Igsf9b	45	53	1.2	69	297	4.3
Gm24357	10	2	0.2	37	79	2.1
Eldr	112	117	1.0	157	348	2.2
